# Patient-specific deep offline artificial pancreas for blood glucose regulation in type 1 diabetes

**DOI:** 10.1016/j.smhl.2026.100633

**Published:** 2026-01-21

**Authors:** Yixiang Deng, Kevin Arao, Christos S. Mantzoros, George Em Karniadakis

**Affiliations:** a Department of Computer and Information Sciences, University of Delaware, Newark, DE 19716, USA; b Department of Medicine, Beth Israel Deaconess Medical Center, Harvard Medical School, Boston, MA 02215, USA; c VA Boston Healthcare System, Harvard Medical School, Boston, MA 02215, USA; d Division of Applied Mathematics, Brown University, Providence, RI 02912, USA

**Keywords:** Artificial pancreas, Type 1 diabetes, Physical exercise, Wearable devices, Offline reinforcement learning, Digital twin

## Abstract

Due to insufficient insulin secretion, patients with type 1 diabetes mellitus (T1DM) are prone to blood glucose fluctuations ranging from hypoglycemia to hyperglycemia. While dangerous hypoglycemia may lead to coma immediately, chronic hyperglycemia increases patients’ risks for cardiorenal and vascular diseases in the long run. In principle, an artificial pancreas – a closed-loop insulin delivery system requiring patients to manually input insulin dosage according to the upcoming meals – could supply exogenous insulin to control the glucose levels and hence reduce the risks from hyperglycemia. However, insulin overdosing in some type 1 diabetic patients, who are physically active, can lead to unexpected hypoglycemia beyond the control of the common artificial pancreas. Therefore, it is important to take into account the glucose decrease due to physical exercise when designing the next-generation artificial pancreas. In this work, we develop a framework integrating systems biology-informed neural networks (SBINN), deep reinforcement learning (RL) algorithms, and T1DM data collected from wearable devices, to automate insulin dosing for patients. In particular, we build patient-specific computational models using SBINN to mimic the glucose-insulin dynamics for a few patients from the dataset, by simultaneously considering patient-specific carbohydrate intake and physical exercise intensity. Our patient-specific artificial pancreas, based on two deep RL algorithms, provided better insulin dosage, leading to safer glucose levels compared to those in the original dataset.

## 1. Introduction

Diabetes mellitus is a growing epidemic and its prevalence has been increasing in nearly all countries (Lovic et al., 2020). Global estimates show that the prevalence of adults aged 20–79 years is 8.8% in 2015, and it is predicted to rise to 10.8% in 2040 (Ogurtsova et al., 2017). In the US, according to the Centers for Disease Control and Prevention (CDC), a total of 34.2 million people have diabetes or 10.5% of the US population in 2018 (Prevention, 2020). The most common forms of diabetes are type 1 diabetes mellitus (T1DM) and type 2 diabetes mellitus (T2DM). In 2016, US data showed T1DM and T2DM accounted for approximately 6% and 91% of all cases of diagnosed diabetes, respectively (Bullard et al., 2018). T1DM is due to autoimmune Beta cell destruction, which usually leads to absolute insulin deficiency. On the other hand, T2DM is due to progressive loss of adequate Beta cell insulin secretion with associated insulin resistance (Care, 2022; Skyler et al., 2017). Glucose is integral to energy consumption as it serves as a primary metabolic fuel. Under normal physiology, in a fasting state, there is a basal insulin secretion to help match hepatic gluconeogenesis to maintain a blood glucose target between 70 and 130 mg/dl. After a meal, there is a rise in blood glucose levels resulting in a concomitant increase in the insulin secretion from the pancreas (Nakrani et al., 2022). The major effects of insulin on glucose metabolism are the following: (a) increases glucose transport across the cell membrane in adipose tissue and muscle, (b) increases glycolysis in muscle and adipose tissue, (c) stimulates glycogenesis and inhibits glycogenolysis in muscle, and liver, and (d) inhibits gluconeogenesis in the liver (Newsholme & Dimitriadis, 2001). A few hours after the meal, as the blood glucose concentration falls, glucagon is secreted to release glucose back into the blood which decreases glucose fluctuations. The main goal of treatment, especially for T1DM patients, is to mimic this physiologic insulin secretion by providing appropriate basal and prandial insulin bolus doses. However, physical exercise often initiates a rise in insulin levels and could potentially cause dangerous hypoglycemia even when the infused insulin is carefully planned. This effect could be amplified in patients with impaired counter-regulation and even a short episode of antecedent hypoglycemia may worsen exercise responses and hence subsequent hypoglycemia (Cockcroft et al., 2020; Davis et al., 2000; Hopkins, 2004). Patients with T1DM are frequently suffering from complications associated with unstable glucose levels when the blood glucose (BG) regulation is not well controlled. According to the Diabetes Control and Complications Trial, better BG control leads to lower HbA1c levels, which finally leads to better outcomes in terms of both microvascular and macrovascular complications (Nathan et al., 2014). Hence, achieving stable glucose levels has been a well-established goal in diabetes management.

Combining a glucose sensor, an insulin infusion device, and a control algorithm, artificial pancreas (AP) is a closed-loop system designed for patients with T1DM to improve their BG regulation, and consequently decrease the risk of diabetic complications (Beck et al., 2019; Cobelli et al., 2011; Ramkissoon et al., 2017; Weisman et al., 2017). While wearable minimally-invasive continuous glucose monitoring (CGM) sensors can provide real-time measurements of blood glucose concentration for days by measuring the glucose concentration in the interstitial fluid (Cappon et al., 2019), commercial products measuring plasma insulin are not widely accessible. The insulin pump, which is an infusion device, provides continuous insulin administration through subcutaneous infusion, instead of needle injections, and greatly improves the quality of life for patients with diabetes (Dwibedi et al., 2022; Ly et al., 2019; Pickup & Keen, 2002; Zhang et al., 2021). Generally, computational models for glucose prediction can be categorized into three different types, i.e., physiology-driven models which are carefully designed by clinicians describing the glucose consumption and production among multiple organs or tissues (Bergman et al., 1979; Hovorka et al., 2004; Man et al., 2014; Visentin et al., 2016), data-driven models which are constructed by training machine learning models based on data collected from glucose monitors (Deng et al., 2021; Lee et al., 2020), and a hybrid approach combining both of them (Woldaregay et al., 2019). In this work, we choose a hybrid model combining a physiology-driven ODE system and deep neural networks with a priority on inferring the hidden dynamics and hidden parameters to build patient-specific in silico glucose-insulin dynamics, in addition to glucose prediction. Specifically, considering the glucose reduction effect due to physical exercises using real-time motion sensors could improve glucose prediction and hence benefit glucose regulation. We were particularly interested in the straightforward and explicit incorporation of physical exercise from a motion sensor and hence chose to use the Roy-Parker model with such functionality, instead of those popular physiology-driven models that only focus on integrating CGM data and insulin pump data, despite their remarkable performance.

Therefore, the next-generation AP in precision medicine will benefit from a holistic design considering important features such as patient-specificity, physical exercises, meal intake. Fortunately, emerging efforts using artificial intelligence (Bent et al., 2021; Deng et al., 2021; Gordon & Stern, 2019; Zhang et al., 2022; Zhu et al., 2022), especially deep neural network-based data-driven machine learning algorithms, provide us with opportunities to predict glucose levels and characterize the complex glucose-insulin dynamics and further enhance the development of control algorithms like reinforcement learning (RL). Unlike supervised learning where a direct input and output mapping is given explicitly, online RL algorithms define the control problem as a Markov Decision Process (MDP) and train an agent, which collects a step-by-step reward through interactions with the environment of interest. The goal of the agent is to respond to the environment changes so that the total reward of the series of responses, namely actions, is maximized by the end of training. Generally, online RL requires a demanding setting where the agent is trained through trial-and-error interactions with a dynamic environment, which can be dangerous for glucose regulation tasks. During these trial-and-error steps, the RL agent may try to optimize for short-term rewards such as glucose levels and end up prescribing too high doses of insulin, which could lead to dangerous hypoglycemia and even unexpected hospitalization when one uses rapid insulin which has a short half-life. Recently, by interacting with in silico simulators, like UVA/Padova T1DMS, several studies developed insulin optimization models based on online RL algorithms for continuous action space, such as Soft Actor-Critic (Fox et al., 2020; Lim et al., 2021), Deep Deterministic Policy Gradient (Zhu et al., 2020), Normalized Advantage Function (Raheb et al., 2022), and Proximal Policy Optimization (Viroonluecha et al., 2022). In general, offline reinforcement learning algorithms are believed to be more efficient than online reinforcement learning because they avoid the interaction with the environment, and it is appreciated that data collections could often be expensive and risky (Fujimoto & Gu, 2021). Unlike online RL, offline RL algorithms utilize only previously collected offline real-world data and do not require additional online interaction with the environment (Levine et al., 2020), providing a promising opportunity for healthcare challenges, where automated drug infusion is necessary (Cai et al., 2023; Emerson et al., 2022). In addition, offline RL algorithms train agents using previously collected data, with no extra interaction with the environment (Kaelbling et al., 1996; Levine et al., 2020), hence minimize the potential risks of harm to diabetic patients. In this study, we focused on prioritizing safety in developing reinforcement learning algorithms in healthcare applications, specifically automating insulin delivery in type 1 diabetes, which is one of the advantages of offline reinforcement learning algorithms. However, the benefits of offline RL also come with some disadvantages, for example, the distributional shift, i.e., while the function approximations might be trained under one distribution, it will be evaluated on a different distribution. To address this challenge, policy constraint methods to offline RL utilize either parameterization or regularization techniques (Wu et al., 2022). Among these approaches, batch-constrained Q learning (BCQ) utilizes policy constraint through parameterization, while the Twin Delayed Deep Deterministic Policy Gradient algorithm with behavior cloning (TD3+BC) achieves a similar outcome through straightforward regularization. In the present work, we apply these two representative offline RL algorithms to optimize the insulin dosage at the patient-specific level.

**Fig. 1.**
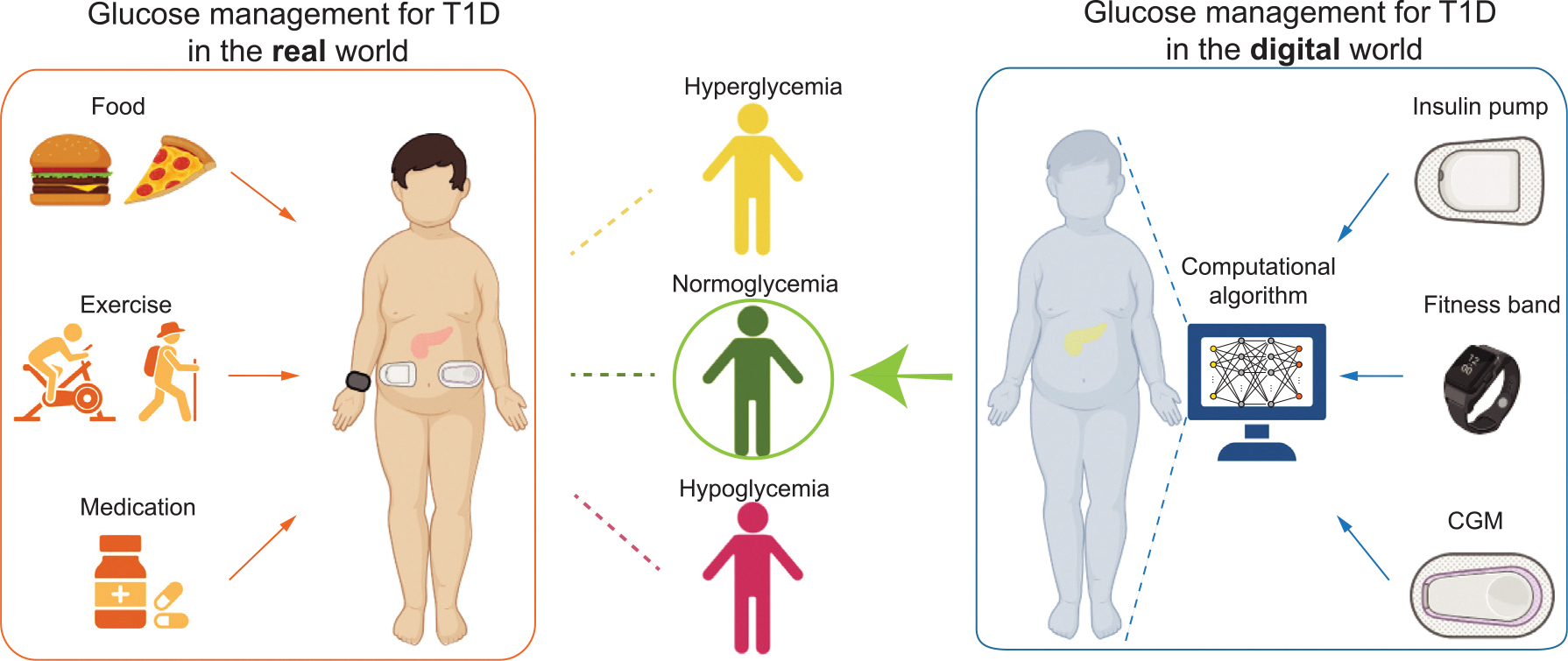
Motivation of digitize blood glucose management in type 1 diabetes. With interaction to the real world, T1DM patients experience fluctuating blood glucose from hypoglycemia (BG levels lower than 70 mg/dl, 80 mg/dl if measured by CGM), normoglycemia (BG levels between 80 and 180 mg/dl), and hyperglycemia (BG levels greater than 180 mg/dl). In the digital world, a digital twin mimicking the glucose-insulin dynamics can be created and optimized using computational algorithm with real-world inputs, i.e., insulin pumps provided exogenous insulin along with carbohydrate intakes, fitness bands provided the exercise intensity, and CGM provided real-time glucose levels. BG, blood glucose. CGM, continuous glucose monitoring. Part of the image is created using Biorender.

Motivated by the features highlighted for next-generation AP design, we propose a novel framework to design a patient-specific artificial pancreas using up-to-date hardware and software technologies with digital twins (Fig. 1). By simultaneously considering patient-specificity, meal intakes, insulin infusion, and most importantly physical exercise, we build and optimize patient-specific glucose levels using a system of ordinary differential equations (ODE) developed by Roy and Parker (Roy & Parker, 2007), time-dependent systems biology informed neural networks (SBINN) (Yazdani et al., 2020), wearable sensor data from the OhioT1DM dataset (Marling & Bunescu, 2020) and two offline RL algorithms. Specifically, when training the time-dependent SBINN, we implement self-adaptive weight to automatically adjust the coefficient of each loss term in the loss function (McClenny & Braga-Neto, 2020), and obtain the dynamics of hidden states and hidden parameters. We design the offline agents such that they learn the insulin dosage with only a short sequence of past glucose levels without any meal or exercise announcements, which significantly advances the step towards an authentic closed-loop system for artificial pancreas design.

## 2. Methods

### 2.1. Framework of the study

**Fig. 2.**
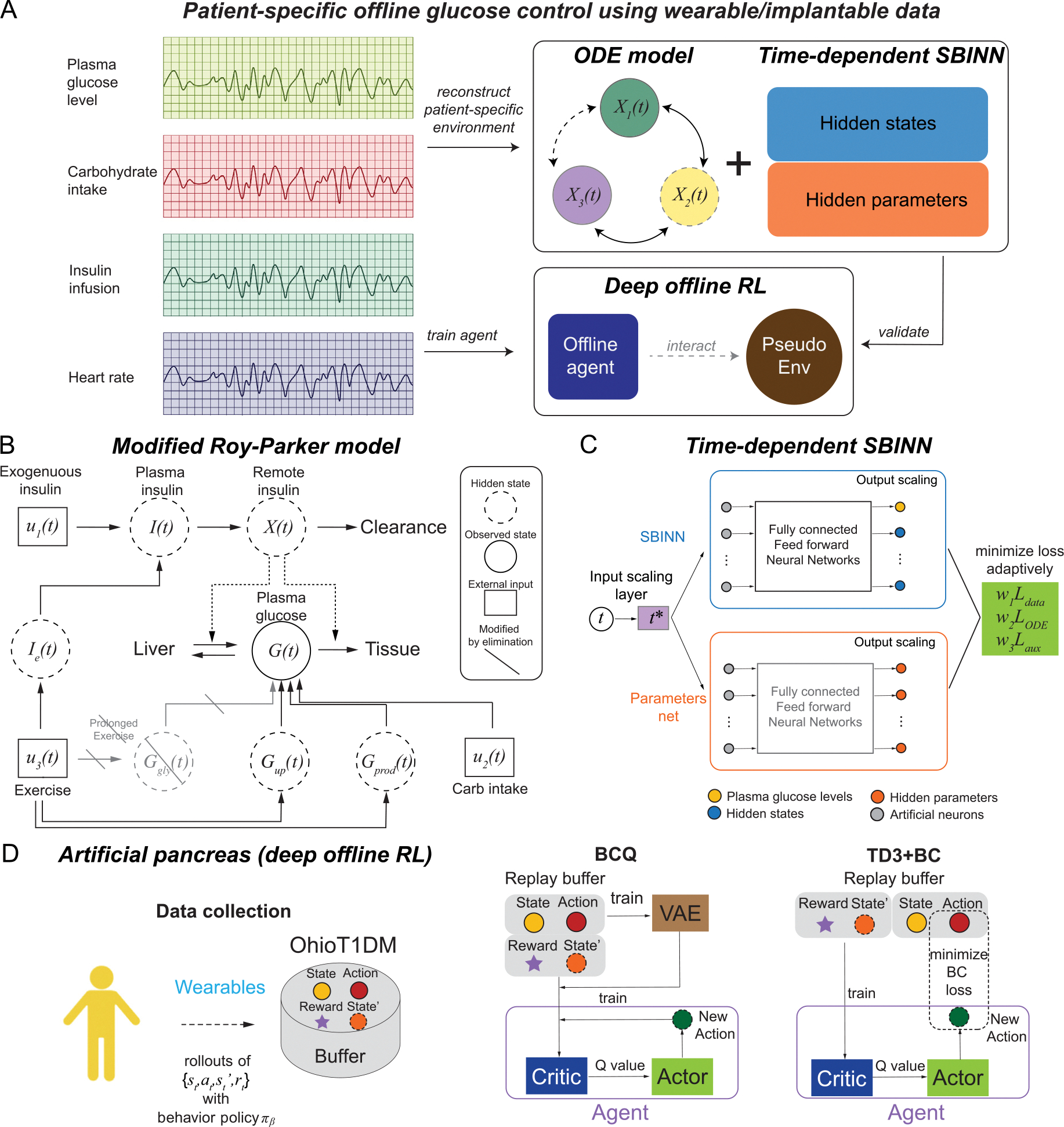
Overview of patient-specific deep offline reinforcement learning for blood glucose regulation. (**A**) Schematic of the framework proposed. Data collected from wearable or implantable devices, such as plasma glucose levels, carbohydrate intakes, insulin infusion and heart rates, were used in two ways. Firstly, the data is applied to reconstruct patient’s metabolic environment, represented by a ODE model and systems biology informed neural networks (SBINN). Meanwhile, the data is also used to train an offline agent composed of neural networks with reinforcement learning algorithms. The reconstructed environment is used to validate the trained offline agent. Part of the image is created using Biorender. (**B**) The compartment diagram of an ODE model, namely modified Roy-Parker model, captures the intrinsic relationships among the observed state G and other hidden state variables, considering external inputs like intake of carbohydrates and intensity of physical exercise. (**C**) Schematic of the time-dependent systems biology informed neural networks (*SBINN*) with self-adaptive weights proposed to learning the dynamics of hidden states and the hidden model parameters. (**D**) Schematic of artificial pancreas design using deep offline reinforcement learning algorithms, BCQ and TD3+BC, in this study. A buffer derived from the OhioT1DM dataset was used to generate replay buffers in both algorithms. An agent represented by a deep neural networks was then trained using merely n plasma glucose levels before time t (t included) to provide the insulin dosage at time t+1. While BCQ extends the online deep Q-learning algorithm to offline with a VAE, TD3+BC extend the online TD3 algorithm to offline with a regularization term to achieve behavior cloning.

In this work, we developed a computational framework to design a patient-specific automated insulin delivery system for six patients with type 1 diabetes using patient-specific data from the OhioT1DM dataset. The OhioT1DM dataset contains eight-week continuous glucose monitoring, insulin, physiological sensor, and self-reported life-event data for 12 patients with type 1 diabetes, among which 6 patients participated in the 2018 cohort and the other 6 patients in the 2020 cohort. A workflow of this framework is shown in Fig. 2A. We first implemented systems biology informed neural networks (SBINN) on the OhioT1DM dataset for parameter inference of the patient-specific Roy-Parker model (Roy & Parker, 2007), based on historical records of total exogenous insulin (bolus insulin and basal insulin), carbohydrate intakes, heart rate, and CGM measured glucose level. We then trained a deep offline RL neural network to build a patient-specific automated insulin delivery system for two representative patients in the OhioT1DM dataset. The final optimized agent, represented by deep neural networks, can serve as the patient-specific artificial pancreas, leading to a better insulin dosage scheme for the patient.

### 2.2. Dataset

#### Dataset overview.

All patients in the OhioT1DM were on insulin pump therapy with continuous glucose monitoring (CGM) throughout the 8-week data collection period. Since the 8-week data was split into training data (first 7 weeks)and testing data (the final 8th week) with no clear instruction to enable an accurate merge of data, we only used the first 7 weeks of training data. Based on the form of the ODE model, i.e., the Roy-Parker model, we selected from the OhioT1DM dataset the following historical measurements: (1) the CGM blood glucose level, (2) insulin doses, both bolus insulin and basal insulin, (3) self-reported meal times and the amounts of carbohydrate intakes, and (4) the heart rate. We note that only the data of those 6 patients in the 2018 cohort is used in our analysis, due to the lack of heart rate monitoring in the 2020 cohort.

#### Data preprocessing.

The exogenous insulin is calculated as the sum of basal insulin and bolus insulin at each moment. According to the user manual of the insulin pumps, i.e., Medtronic 530G and 630G, while the basal insulin $u_{1\text{bolus}}$ is given at a rate and is provided explicitly in the electronic health record, bolus insulin $u_{1\text{bolus}}$ is a one-time dose and can be released into the blood stream using different mode, i.e., “normal”, “normal dual”, “square” and “square dual”. Additionally, we also consider “temp basal” insulin $u_{1\text{temp\_basal}}$, which overrides the basal insulin set previously. Given limited information for the exact releasing process of these different mode in the OhioT1DM dataset, we assume the conversion formula based on literature (Heinemann, 2009) and the user guides of the corresponding commercial insulin pumps (Medtronic Diabetes, 2016, 2018). The mathematical formula of total insulin infusion rate $u_{1}(t)$ is given as follow.

(1) $$u_{1}(t)=u_{1\text{basal}}(t)+\left(1-{𝟙}_{\text{temp}\;\text{basal}}\right)u_{1\text{bolus}}(t)+{𝟙}_{\text{temp}\;\text{basal}}u_{1\text{temp\_basal}}(t)$$

where ${𝟙}_{\text{temp}\;\text{basal}}$ denotes that the “temp basal” option is available at time t. Depending different mode, $u_{1\text{bolus}}(t)$ is computed as follow,

(2) $$u_{1\text{bolus}}(t)=\left\{\begin{array}{ll} \frac{x}{10} & \text{mode}=\text{“normal”}\\ \frac{x}{t_{\text{end}}-t_{\text{start}}} & \text{mode}=\text{“square”}\\ {𝟙}_{t_{\text{start}}\leq t\leq \frac{t_{\text{start}}+t_{\text{end}}}{2}}\frac{0.5x}{10}+{𝟙}_{\frac{t_{\text{start}}+t_{\text{end}}}{2}<t\leq t_{\text{end}}}\frac{0.5x}{0.5\left(t_{\text{end}}-t_{\text{start}}\right)} & \text{mode}=\text{“normal}\;\text{dual”}\\ {𝟙}_{t_{\text{start}}\leq t\leq \frac{t_{\text{start}}+t_{\text{end}}}{2}}\frac{0.5x}{0.5\left(t_{\text{end}}-t_{\text{start}}\right)}+{𝟙}_{\frac{t_{\text{start}}+t_{\text{end}}}{2}<t\leq t_{\text{end}}}\frac{0.5x}{10} & \text{mode}=\text{“square}\;\text{dual”} \end{array}\right.$$

where x denotes single-dose bolus insulin, 10 approximates the releasing time in minutes for bolus insulin at “normal” mode, $t_{\text{end}}$ denotes the moment the corresponding mode ends and $t_{\text{start}}$ denotes the moment the corresponding mode starts are given in the electronic health record. In “dual” mode, the bolus dose is evenly divided in two halves and released with two modes sequentially.

The glucose consumption rate $u_{2}$ due to meal intakes is computed by the amount of meal carbohydrate using an exponential decay function (Yazdani et al., 2020) as follows,

(3) $$u_{2}(t)=0.0083\sum_{j=1}^{N}m_{j}exp\left(0.0083\left(t_{j}-t\right)\right),$$

where $m_{j}$ gram of carbohydrate intake is recorded at $t_{j},\;N$ is the total number of meals, and the decaying constant is derived from the glucose-insulin study case in Yazdani et al. (2020), based on the work of Sturis et al. (1991).

Several studies have demonstrated the feasibility of using a target heart rate as a tool for exercise prescription (Karvonen & Vuorimaa, 1988; Porcari et al., 2015; Thomson et al., 2019). We specifically use heart rate collected from the fitness band to quantify the exercise intensity, represented by the percentage of ${VO}_{max}^{2}\left({PVO}_{max}^{2}\right)$ with an empirical formula as follows (Lepretre et al., 2004),

(4) $$\begin{array}{l} PVO_{2}^{max}(t)\;=0.888HR-71.91,\\ \;u_{3}(t)\;=PVO_{2}^{max}(t)-8,\;\text{if}\;u_{3}(t)>0\;\text{else}\;0, \end{array}$$

where 8 denotes the average $PVO_{2}^{\text{max}}$ for a person at the basal state (Roy & Parker, 2007). For missing heart rate values, we apply data imputation using linear interpolation with adjacently available heart rates. After converting all time sequences into the same time resolution, we trim the time sequences of different sources such that the starting time stamp of the final data is the latest of all time sequences and the ending time stamp of the final data is the earliest of all time sequences. We also further smooth the data using a rolling window and generate a coarse-grained dataset, where the sampling interval is 1 h between neighboring time points. Fig. S2 shows the processed insulin infusion $u_{1}(t)$, carbohydrate intake $u_{2}(t)$ and exercise intensity $u_{3}(t)$ for 6 patients in the OhioT1DM dataset. Table S2 shows the preprocessed dataset for 6 patients.

### 2.3. Systems biology informed neural networks (SBINN) with the Roy-Parker model

#### Roy-Parker model.

With the aim of developing a robust closed-loop insulin delivery system under changing physiological conditions, Roy and Parker developed a model that can predict blood glucose levels at rest and during physical exercises (Roy & Parker, 2007). Since the patients participated in OhioT1DM only performed sporadic and light physical activities, we modified the ODE system in Roy and Parker (Roy & Parker, 2007) by omitting the $G_{gly}(t)$ term representing the decline of the glycegenolysis rate during prolonged exercise due to the depletion of liver glycogen stores (Fig. 2B). The resulting ODE system for the 6 state variables $\left[I,X,G,G_{\mathrm{prod}},G_{\mathrm{up}},I_{e}\right]$ is shown in Eqs. (5)–(10),

(5) $$\frac{dI(t)}{dt}=-nI\left(t\right)+p_{4}u_{1}\left(t\right)-I_{e}\left(t\right);\;I\left(0\right)=I_{b}=\frac{p_{4}}{n}u_{1b};$$

(6) $$\frac{dX(t)}{dt}=-p_{2}X\left(t\right)+p_{3}\left[I\left(t\right)-I_{b}\right];\;X(0)=0;$$

(7) $$\frac{dG(t)}{dt}=-p_{1}\left[G(t)-G_{b}\right]-X(t)G(t)+\frac{W}{Vol_{G}}G_{prod}(t)-\frac{W}{Vol_{G}}G_{up}(t)+\frac{u_{2}(t)}{Vol_{G}};\;G(0)=G_{b};$$

(8) $$\frac{dG_{prod}(t)}{dt}=a_{1}PVO_{2}^{max}(t)-a_{2}G_{prod}(t);\;G_{prod}(0)=0;$$

(9) $$\frac{dG_{up}(t)}{dt}=a_{3}PVO_{2}^{max}(t)-a_{4}G_{up}(t);\;G_{up}(0)=0;$$

(10) $$\frac{dI_{e}(t)}{dt}=a_{5}PVO_{2}^{max}(t)-a_{6}I_{e}(t);\;I_{e}(0)=0.$$

This ODE system captures the exercise-induced dynamics of plasma insulin concentration I(t), remote insulin concentration X(t), the plasma glucose level G(t), exercise-induced hepatic glucose production $G_{\mathrm{prod}}(t)$, exercise-induced glucose uptake $G_{\mathrm{up}}(t)$, exercise-induced insulin removal from the circulatory system $I_{e}(t)$, exogenous infusion $u_{1}(t)$, and external glucose uptake $u_{2}(t)$. The instant parameters $I_{b}$ and $G_{b}$ represent the basal plasma insulin and glucose concentrations, respectively. Given the multi-scale nature of the glucose levels in a month-long observation, we found that our algorithm learns better the glucose dynamics when we allow the parameters in the ODE to vary over time. Table S1 shows the nomenclature, physiological meaning and reference values of patient-specific parameters to be inferred in the ODE. The ranges of the parameters were set to $\left[0.2\cdot x_{\mathrm{ref}},1.8\cdot x_{\mathrm{ref}}\right]$, where $x_{\mathrm{ref}}$ represents the corresponding reference value of the variable in Table S1.

#### Systems biology informed neural networks (SBINN).

Yazdani et al. developed a general framework, namely systems biology informed neural networks (SBINN), to solve all states described by a system of ODEs as well as simultaneously estimating the parameters involved (Yazdani et al., 2020). Fig. 2C shows the structure of SBINN, which is sequentially composed of an input-scaling layer to allow input normalization for the robust performance of the neural networks, a feature layer marking different patterns of state variables in ODEs and the output-scaling layer to convert normalized state variables back to physical units. By effectively adding constraints derived from the ODE system to the optimization procedure, SBINN is able to simultaneously infer the dynamics of unobserved species, external forcing, and the unknown model parameters.

Given the measurements of $y_{1},y_{2},\ldots ,y_{M}$ at times $t_{1},t_{2},\ldots ,t_{N^{\mathrm{data}}}$, SBINN enforces the network to satisfy the ODE of interest at the time point $\tau_{1},\tau_{2},\ldots ,\tau_{N^{\mathrm{ode}}}$. To solve an initial value problem or a final value problem for ODE which encoded physics, one needs to compute the solution such that it satisfies the initial and/or final values as well as minimizing the residue of ODE. For data-driven approaches like physics-informed neural networks or SBINN, one needs to additionally minimize the difference between the observed data and the neural network used to approximate the state variable, which generates the observed data. Hence, SBINN defines the total loss as a function of both the parameters of the neural networks, denoted by θ and parameters of the ODE, denoted by p.

(11) $$ℒ(\theta ,p)={ℒ}^{\mathrm{data}}(\theta )+{ℒ}^{\mathrm{ode}}(\theta ,p)+{ℒ}^{\mathrm{aux}}(\theta ),$$

where ${ℒ}^{\mathrm{data}}$ is associated with the M sets of observations of the state variables y in the ODE to address the data-driven loss; ${ℒ}^{\mathrm{ode}}$ represents the residue of ODE to be minimized; ${ℒ}^{\mathrm{aux}}$ is defined to satisfy the initial value and/or final value. The final step of SBINN is to infer the neural network parameters θ as well as the unknown ODE parameters p simultaneously by minimizing the aforementioned loss function via gradient-based optimizers Kingma and Ba (2014).

In this work, our ODE of interest is the modified Roy-Parker model presented above and we substantiated the terms in ODE as follows. The known observed state variable y is the CGM measured glucose record in the OhioT1DM dataset, i.e., G(t), which is used for minimizing the data loss, ${ℒ}^{\mathrm{data}}$. We used ${ℒ}^{\mathrm{ode}}$ to minimize the residual terms in the ODE, shown in Eqs. (5)–(10). Following Yazdani et al. (2020), we imposed the initial condition as the auxiliary loss ${ℒ}^{\mathrm{aux}}$. To improve the training of SBINN and speed up the convergence, we implemented self-adaptive weights over each iteration on the weights of each loss terms (McClenny & Braga-Neto, 2020). The self-adaptive weighted loss ${ℒ}^{\prime }$ is as follows,

(12) $${ℒ}^{\prime }\left(\theta ,p,\lambda_{o},\lambda_{a}\right)={ℒ}^{\mathrm{data}}(\theta )+\lambda_{o}{ℒ}^{\mathrm{ode}}(\theta ,p)+\lambda_{a}{ℒ}^{\mathrm{aux}}(\theta ),$$

where $\lambda_{o}$ and $\lambda_{a}$ are trainable, non-negative self-adaptation weights associated to the ODE loss term and auxiliary loss term, respectively. Hence, the objective of the training of neural networks is updated to

(13) $$\underset{\theta }{min}\underset{\lambda_{o},\lambda_{a}}{max}{ℒ}^{\prime }\left(\theta ,p,\lambda_{o},\lambda_{a}\right).$$

The update rules for the self-adaptive weights are given by,

(14) $$\begin{array}{rr} & \lambda_{o}^{k+1}=\lambda_{o}^{k}+\rho_{o}^{k}\nabla_{\lambda_{o}}{ℒ}^{\prime }\left(\theta ,p,\lambda_{o},\lambda_{a}\right),\\ & \lambda_{a}^{k+1}=\lambda_{a}^{k}+\rho_{a}^{k}\nabla_{\lambda_{a}}{ℒ}^{\prime }\left(\theta ,p,\lambda_{o},\lambda_{a}\right). \end{array}$$

where $\rho_{o}^{k}$ and $\rho_{a}^{k}$ denotes the learning rates for the corresponding weights, which was set to be $\rho_{o}^{k}=\rho_{a}^{k}$ in this work.

### 2.4. Offline reinforcement learning

We formulated the RL problem for glucose regulation into an MDP as follows. The state $S_{t}$ is defined by a continuous sequence of glucose levels in a time window w, i.e., $S_{t}=\left[G_{t-w+1},G_{t-w+2},\ldots ,G_{t}\right]$, specifically, we used w=5 to allow the model to learn enough glucose dynamics. The action $A_{t}$ is defined as the insulin infusion rate at time t, i.e., $u_{1}(t)$. We fixed the glucose infusion rate $u_{2}$ and exercise intensity $u_{3}$ from the OhioT1DM dataset and trained the agent to provide the optimal exogenous insulin infusion rate $u_{1}^{opt}$ for a month. The return function $r_{t}$ and the reward function R of a complete episode are defined as follows,

(15) $$r_{t}=\left\{\begin{array}{ll} -{\left(\frac{G_{t}-G_{\mathrm{target}}}{G_{\mathrm{target}}}\right)}^{2}, & G_{min}\leq G_{t}\leq G_{max};\\ -10, & \mathrm{otherwise.} \end{array}\right.$$

(16) $$R=\sum_{i=0}^{T_{\mathrm{end}}}\gamma^{i}r_{i},$$

where we set the target glucose level to be 120 mg/dl, $G_{\min}=80,\;G_{\max}=180$. We have imposed a constraint on the maximum insulin infusion rate of 90 mU/min (which is derived from the highest observed infusion rate in the OhioT1DM dataset, based on these six patients) in our code implementation to avoid hypoglycemia induced by insulin overdosage regardless of the weight of patients. We implemented two offline RL algorithms, i.e., BCQ and TD3+BC. Both RL algorithms were tested on an NVIDIA RTX A6000 GPU for 10000 episodes, taking about 24 h in physical runtime. In both methods, the optimal hyperparameter sets were determined by grid search.

#### Batch-constrained Q learning (BCQ).

Herein, we implemented the batch constrained Q-learning (BCQ) algorithm (Fujimoto et al., 2019) to optimize personalized insulin dosage for each patient from OhioT1DM (Fig. 2D). In BCQ, a buffer dataset is first collected by some behavior policy $\pi_{\beta }$, before the training starts. Specifically, we generated a buffer by sampling from the OhioT1DM dataset and generate the states, i.e., glucose levels, carbon intakes and physical exercises, along with the action denoted by total exogenous insulin at a specific time point, i.e., a sum of the bolus insulin and basal insulin, and the corresponding returns depending on the resulting glucose levels. Afterwards, the agent represented by a deep neural networks is trained with the RL algorithm (Fujimoto et al., 2019). Finally, a policy outperforming the behavioral policy $\pi_{\beta }$ is deployed in the patient-specific artificial pancreas. By restricting the action space in order to force the agent towards behaving close to a subset of the given data, BCQ is able to learn successfully without interacting with the environment by considering extrapolation error. The details of the BCQ algorithm are shown in Algorithm 1. Following Fujimoto et al. (2019), we implemented a fully-connected feed forward neural network for the Q-networks to represent the agent’s action, i.e., the insulin dosage and the variational autoencoders (VAE), which is defined by two networks, an encoder network E(s,a) and a decoder network D(s,z), where z is the latent vector, to obtain the consequent glucose response. The hyper-parameters used in this work can be found in Table S3.

*TD3*+*BC.* We also test the performance of another offline reinforcement learning algorithm, i.e., TD3+BC (Fujimoto & Gu, 2021), which combines Twin Delayed Deep Deterministic policy gradient (TD3) algorithm (Fujimoto et al., 2018) with behavior cloning (BC). Based on Deep Deterministic Policy Gradient (DDPG) (Lillicrap et al., 2015), TD3 improves performance by (1) learning two Q-values, and uses the smaller of the two Q-values to form the targets in the Bellman error loss functions, (2) updating the policy and target networks less frequently than the Q-values and (3) adding noise to the target action, to avoid the policy to exploit Q-value errors by smoothing out Q along changes in action. By adding a single adjustment to the policy update process of the TD3 algorithm with the following policy update rule

(17) $$\pi ={argmax}_{\pi^{\prime }}E_{(s,a)∼D}\left[\lambda Q\left(s,\pi^{\prime }(s)\right)-{\left(\pi^{\prime }(s)-a\right)}^{2}\right],$$

where π denotes the RL policy, D denotes the distribution of state s and action a pair from offline buffer, $\lambda =\frac{\alpha }{\frac{1}{N}\sum_{\left(s,a\right)}|Q(s,a)|}$ is a hyperparameter. The details of the TD3+BC algorithm are shown in Algorithm 2. For offline buffer generation, we reused the buffer for each patient generated in BCQ. For neural network architectures of actors and critics, we followed the implementation of Fujimoto et al. (2018). The hyper-parameters and architecture details used in this work can be found in Table S4.

## 3. Results

To build a surrogate environment for our agent to interact with, we first performed patient-specific parameter inference using SBINN with a system of ODE developed by Roy and Parker (2007). The primary step of parameter inference for a system of ODE is to examine its identifiability. After obtaining the patient-specific parameters of the ODE, which is essential to reconstruct the dynamics of the state variables, we developed a patient-specific offline RL algorithm to learn an optimal planning for the external insulin infusion for two representative patients, which helped them decrease the risk of hypoglycemia and hyperglycemia, respectively.

### ODE identifiability of the Roy-Parker model

We first performed structural identification on the ODE for the set of parameters

$$\left\{n,{Vol}_{G},p_{1},p_{2},p_{3},p_{4},a_{1},a_{2},a_{3},a_{4},a_{5},a_{6},W,u_{1b}\right\}$$

appeared in Eqs. (5)–(10). Although most of the ODE parameters are not readily available in the OhioT1DM dataset, there are several parameters that are practically available for patients. For example, the patient’s body weight (W) can be measured practically but is not available in OhioT1DM. While $u_{1b}$, the exogenous insulin infusion rate to maintain basal plasma insulin, is not available in the OhioT1DM, but it could be inferred from the mode of the exogenous insulin profile of the specific patient. We considered the identifiability of the aforementioned parameters under different scenarios depending on whether W and $u_{1b}$ are known for the given patient. Table 1 suggests that when W and $u_{1b}$ are both known, other parameters are either globally identifiable or locally identifiable. We adopted this scenario in the following analysis by assuming W = 60 kg and patient-specific $u_{1b}$ to construct the patient-specific model by inferring a subset of the aforementioned parameters $\left\{n(t),{Vol}_{G}(t),p_{1}(t),p_{2}(t),p_{3}(t),p_{4}(t),a_{1}(t),a_{2}(t),a_{3}(t),a_{4}(t),a_{5}(t),a_{6}(t)\right\}$ for one-month period.

### Patient-specific parameter inference and kinetics reconstruction using OhioT1DM

In the OhioT1DM dataset, we assumed the patient’s weight is 60 kg, i.e., W = 60 kg. We note that the weight parameter can be absorbed in ${Vol}_{G}$, which will remain within a reasonable range after rescaling to accommodate a higher body weight (Ferrannini et al., 1985). We also assume that the initial condition of the ODE system is given by ${\left[I,X,G,G_{\mathrm{prod}},G_{\mathrm{up}},I_{e},PVO_{\max}^{2}\right]}_{t=0}=\left[I_{b},0,G_{b},0,0,0,0\right]$, where $I_{b}$ is estimated from the basal insulin rate and $G_{b}$ is the initial blood glucose level, both of which are patient-specific estimations from the OhioT1DM dataset. We imposed the initial condition of the state variables as the auxiliary loss. We also imposed smoothing using a moving window of 30 data points on the model inputs to speed up the convergence of parameter inference.

**Fig. 3.**
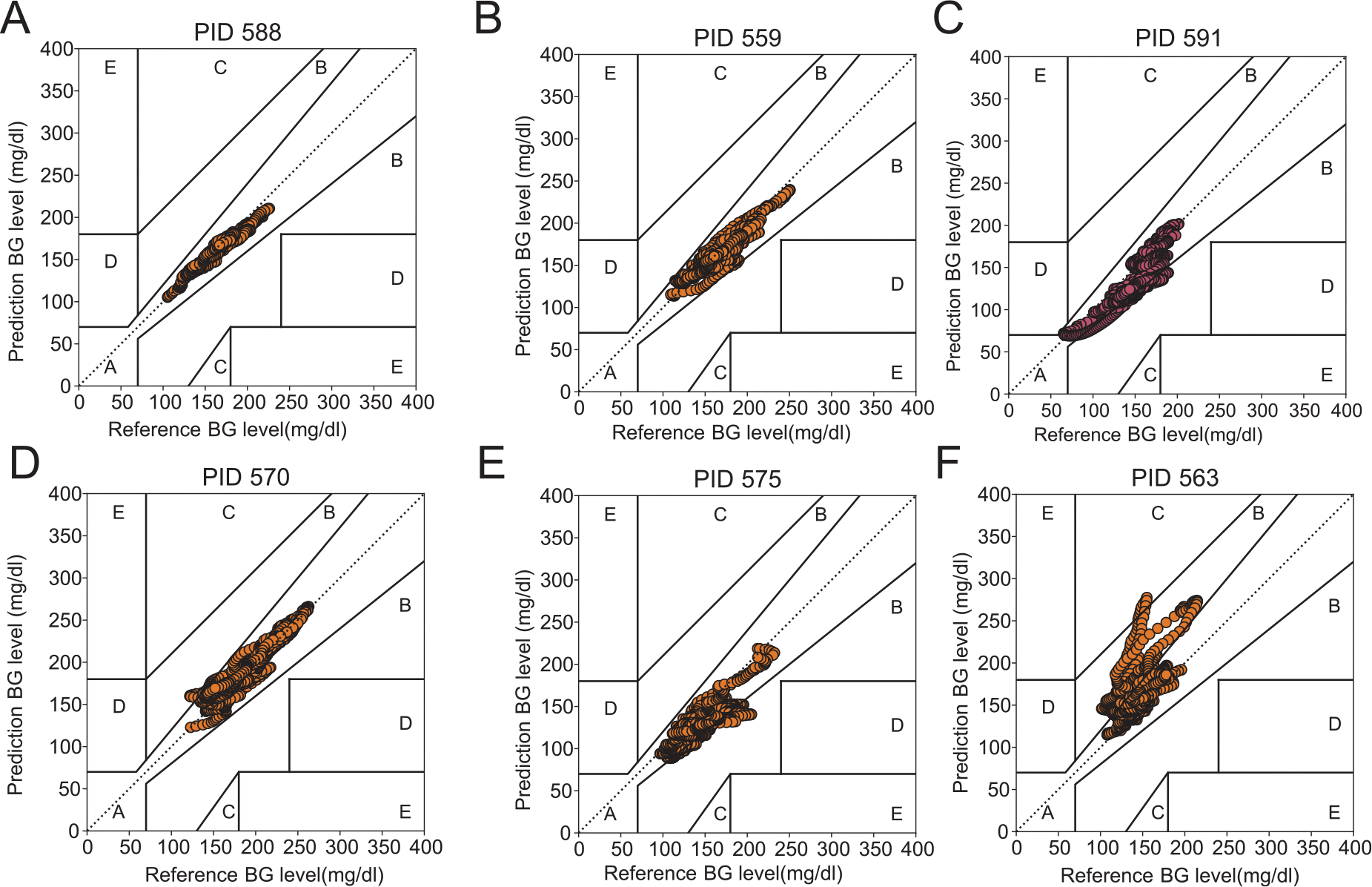
Clarke error grid analysis of glucose levels predicted by time-dependent SBINN in the Roy-Parker model and the reference blood glucose levels in the OhioT1DM dataset for patients in OhioT1DM. Clarke error grid for (**A**) patient with ID 588.(**B**) patient with ID 559. (**C**) patient with ID 591. (**D**) patient with ID 570. (**E**) patient with ID 575. (**F**) patient with ID 563. In each panel, Region A corresponds to those values within 20% of the reference sensor; Region B contains points that are outside of 20% but would not lead to inappropriate treatment; Region C corresponds to those points leading to unnecessary treatment; Region D corresponds to those points indicating a potentially dangerous failure to detect hypoglycemia or hyperglycemia; Region E corresponds to those points that would confuse treatment of hypoglycemia for hyperglycemia and vice versa. Dots in patient 591 are marked with pink given the occurrence of hypoglycemia in the corresponding data, otherwise dots are marked with yellow denoting frequent hyperglycemia occurrence observed in the OhioT1DM dataset. BG denotes blood glucose.

To confirm the accuracy of the inferred parameters, we have plotted the kinetics of both CGM measured BG and BG obtained based on the solution of the system of ODEs. Additionally, we examined the performance of our model with Clarke Error Grid analysis (Clarke et al., 1987), which describes the clinical accuracy of models over the entire range of blood glucose values with clearly labeled domains implying clinical decisions. Fig. 3 shows the Clarke error grid analysis of time-dependent SBINN predicted BG levels, i.e., glucose levels obtained from solving the ODE system using the parameters inferred by SBINN vs. the sensor data collected in OhioT1DM for all six patients. We found that most of the prediction-reference BG pairs for all patients lie in regions A and B, which are helpful for appropriate treatment. Specifically, a time-dependent SBINN method showed high accuracy in five patients (Fig. 3 (A)–(E)), most prediction-reference BG pairs that lie in region A, which are considered clinically accurate. Despite that, a few prediction-reference BG pairs for patient 563 lie in region C; we note that they are mostly in the upper triangle of the grid, which means the prediction BG levels are slightly higher than the reference BG levels, and are not likely to lead to insulin overdose or hypoglycemic event.

**Fig. 4.**
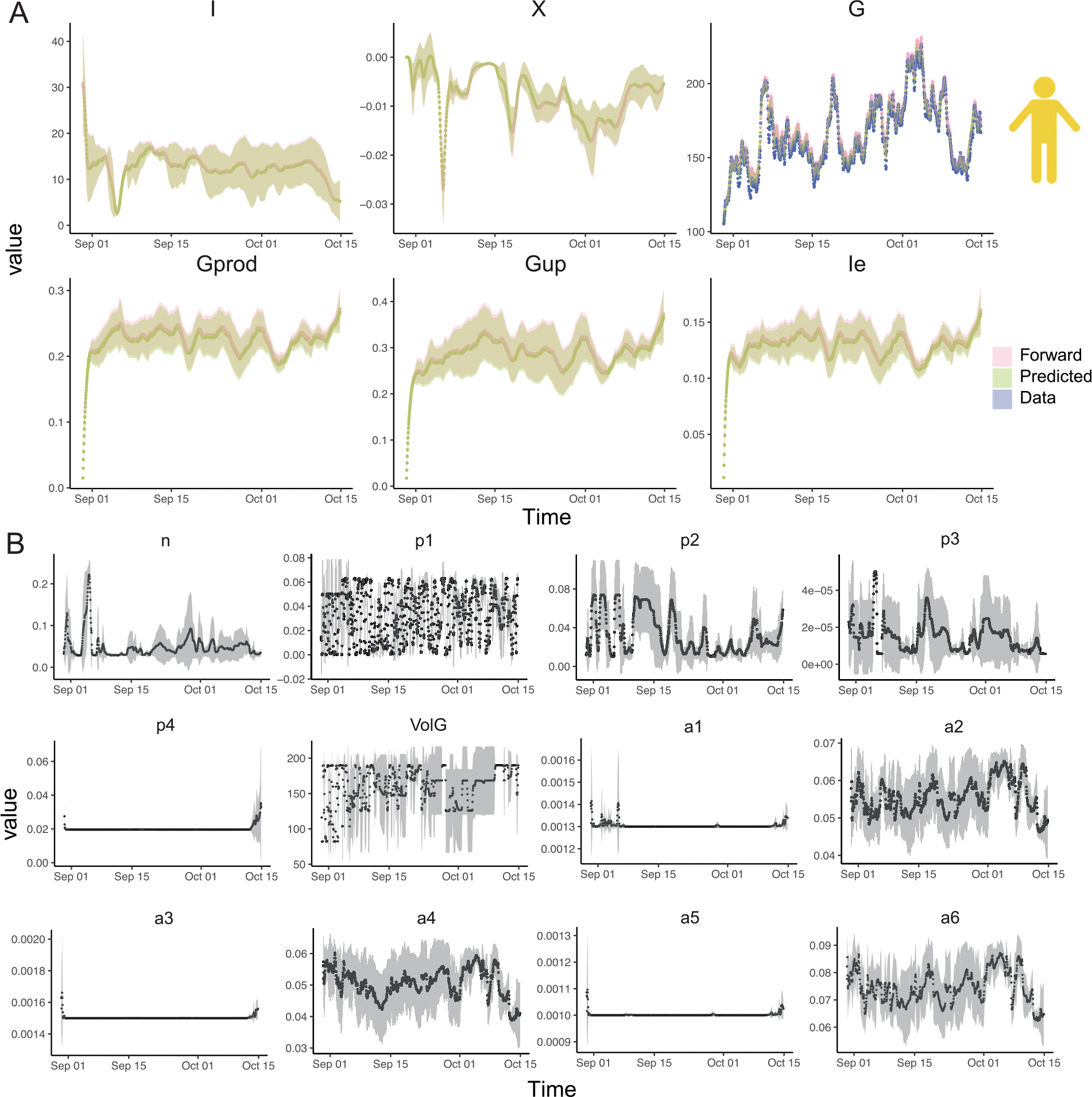
Time-dependent SBINN accurately infers the dynamics of state variables and hidden parameters in the Roy-Parker model for a patient in OhioT1DM with *recurrent hyperglycemia*. (**A**) The trajectory of state variables, plasma insulin level I(t), remote insulin level X(t), plasma glucose level G(t), exercise-induced hepatic glucose production $G_{\mathrm{prod}}(t)$, exercise-induced glucose uptake $G_{\mathrm{up}}(t)$, exercise-induced insulin removal from the circulatory system $I_{e}(t)$. Patient ID 588, shown here, experienced repeated hyperglycemic period, suggested by the plasma glucose levels G repeatedly exceeding 180 mg/dl. The blue solid dots denotes plasma glucose levels collected by CGM. The pink line denotes mean of the forward solution of the ODE from 5 different runs using inferred parameters, with the pink shade denoting the corresponding standard deviation. The green line denotes mean of denotes the predicted values by SBINN from 5 different runs using inferred parameters, with the green shade denoting the corresponding standard deviation. (**B**) The trajectory of patient-specific time-dependent hidden parameters in the Roy-Parker model for patient ID 588. The black dots denote the mean of each hidden parameters from 5 different runs and the gray shade denotes the corresponding standard deviation.

**Fig. 5.**
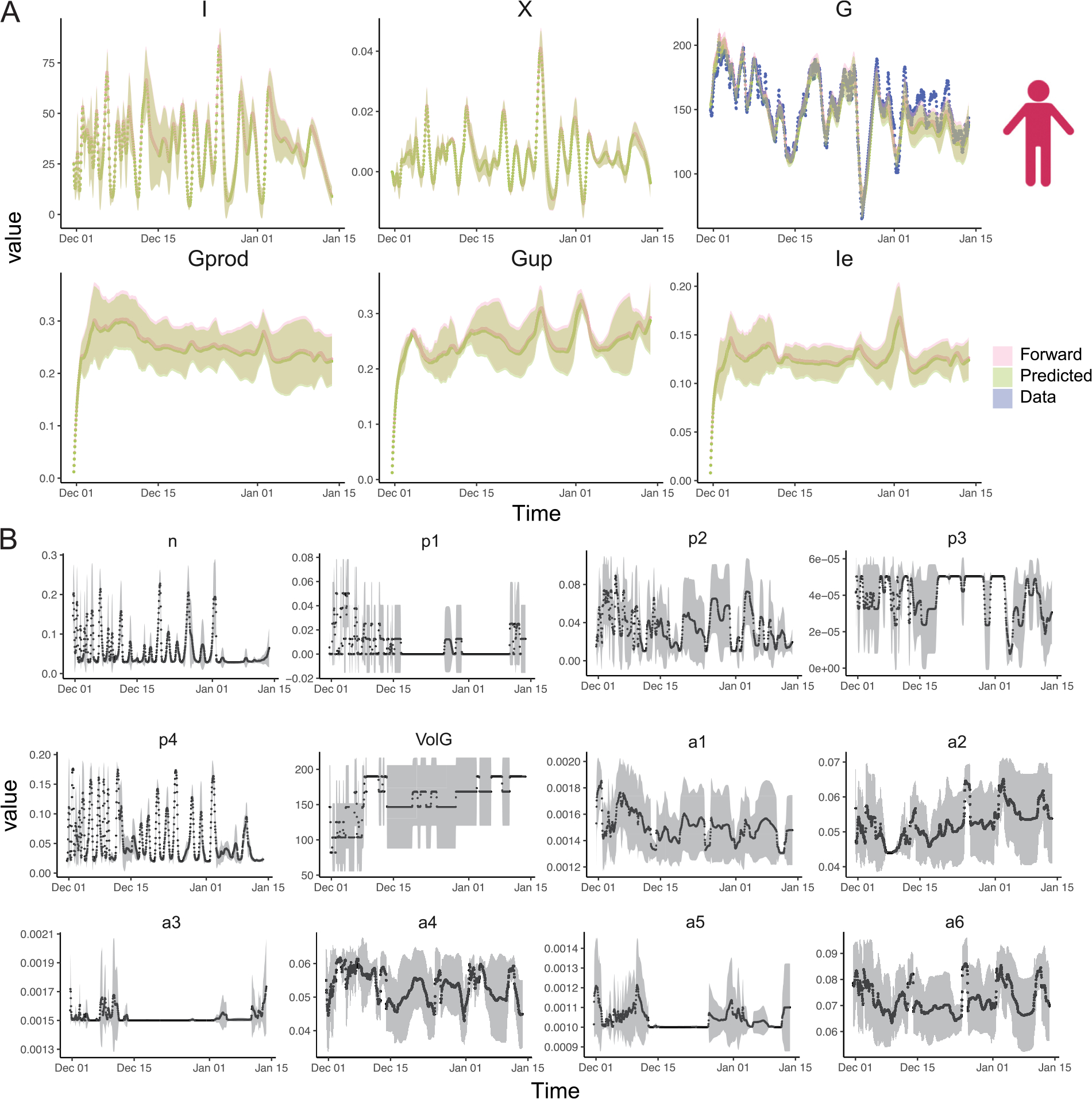
Time-dependent SBINN accurately infers the dynamics of state variables and hidden parameters in the Roy-Parker model for a patient in OhioT1DM with *occasional hypoglycemia*. (**A**) The trajectory of state variables, plasma insulin level I(t), remote insulin level X(t), plasma glucose level G(t), exercise-induced hepatic glucose production $G_{\mathrm{prod}}(t)$, exercise-induced glucose uptake $G_{\mathrm{up}}(t)$, exercise-induced insulin removal from the circulatory system $I_{e}(t)$. Patient ID 591, shown here, experienced a short hypoglycemic period, suggested by the plasma glucose levels went below 80 mg/dl for a short period. The blue solid dots denotes plasma glucose levels collected by CGM. The pink line denotes mean of the forward solution of the ODE from 5 different runs using inferred parameters, with the pink shade denoting the corresponding standard deviation. The green line denotes mean of denotes the predicted values by SBINN from 5 different runs using inferred parameters, with the green shade denoting the corresponding standard deviation. (**B**) The trajectory of patient-specific time-dependent hidden parameters in Roy-Parker model for patient ID 591. The black dots denote the mean of each hidden parameters from 5 different runs and the gray shade denotes the corresponding standard deviation.

Besides accurately predicting glucose levels over time like typical data-driven glucose prediction algorithms, we aim to infer the hidden parameters governing ODE by effectively integrating SBINN and data from wearable sensors, and consequently construct a patient-specific in silico model characterizing glucose-insulin dynamics for subsequent training of offline reinforcement learning agents. The way of determining if the hidden parameters are correctly inferred is by solving the initial value problem and comparing the obtained BG with the BG measured by CGM. Fig. 4 shows the inference of model parameters and hidden kinetics on patient ID 588, who experienced repeated hyperglycemic events during the data collection period. Fig. 5 shows the same inference on another patient ID 591, who experienced a short period of hypoglycemia (glucose level below 80 mg/dl, we adjusted the threshold of hypoglycemia due to an overestimation of glucose levels by CGM (Farrell et al., 2020)) around December 22, 2021. The corresponding results for the other four patients (IDs: 559, 563, 570, 575) can be found in Fig. S3–S6 in the Supplementary Material. Note that the time stamps in OhioT1DM dataset are pre-processed to avoid privacy leakage, hence they do not represent the real collection time. Inspired by observed fluctuation of metabolic reaction rates in oral glucose tolerance test (OGTT) (Yoshino et al., 2022), we adopted a time-varying parameters setting for SBINN to improve the flexibility of our model. Interestingly, while patient 588 and patient 591 followed similar insulin infusion and carb intakes, the only lifestyle difference in the exercise intensity seemingly changed the outcomes of their glucose management (Fig. S2).

We found frequently elevated plasma insulin and remote insulin in patient 591 compared to patient 588 (Fig. 4A and Fig. 5A), suggesting that patient 591 may have a higher risk of developing hyperinsulinemia than patient 588. We also observed that some of the hidden parameters of patient 588 do not fluctuate as significantly over time as those of patient 591 (Fig. 4B and Fig. 5B). These parameters are $p_{4}$, denoting the rate of insulin addition into the plasma from exogenous insulin, $a_{1}$, denoting the rate of exercise-induced hepatic glucose production, $a_{3}$, denoting the rate of exercise-induced glucose uptake, and $a_{5}$, denoting the rate of exercise-induced plasma insulin depletion during the recovery period. Especially, we observed a sudden increase in terms of $a_{5}$ for patient 591 right after the occurrence of exercise-induced hypoglycemia, right before Jan 01. We also found that some parameters are significantly different between patient 591 and patient 588 on average. These parameters are n, denoting the rate of plasma insulin clearance, $p_{3}$, denoting the rate of insulin addition in the remote insulin compartment, and $p_{4}$, denoting the rate of insulin addition into the plasma from exogenous insulin. Interestingly, all these parameters point to the balance of remote insulin and plasma insulin, with n and $p_{3}$ being almost doubled in 591 while $p_{4}$ being frequently higher in 588. These findings together imply that the glucose level fluctuation is a complex process involving multiple organs and tissues, and exercise contributes to the occurrence of hypoglycemia in a more complicated way than merely lowering the plasma glucose level.

Our results suggest that time-dependent SBINN successfully infers the fluctuating hidden kinetics as well as the parameters in all these 6 patients, albeit the inter-patient variability due to different daily routines of physical activities and insulin injection. More importantly, we also observed that time-dependent SBINN were able to perform robustly under 5 different random seeds with the uncertainty band confined to an acceptable range. We specifically emphasized the accuracy of our parameter inference, which helped us reconstruct the dynamics by solving a forward problem with high accuracy, indicated by the good match between pink curves (forward solver) and blue curves (real data collection).

### Offline reinforcement learning

**Fig. 6.**
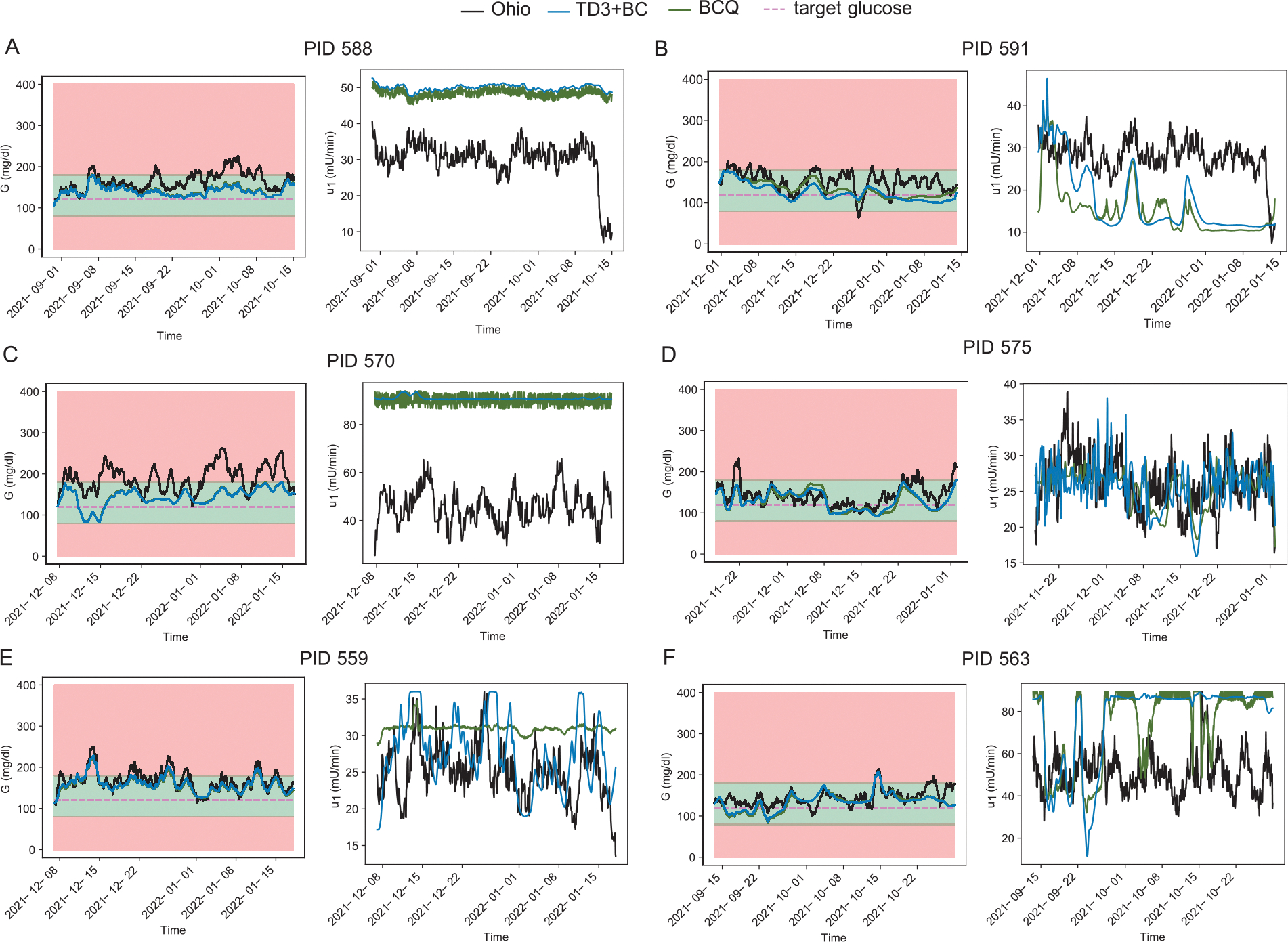
Deep offline reinforcement learning stabilizes plasma glucose level variation for 5 patients in the OhioT2DM 2018 cohort, compared to the archived data in the OhioT1DM. Glucose trajectories (left panels) along with the insulin trajectories (right panels) of RL agents (BCQ, green curve; TD3+BC, blue curve) and original Ohio data (black curve). (**A**) patient with ID 588. (**B**) patient with ID 591. (**C**) patient with ID 570. (**D**) patient with ID 575. (**E**) patient with ID 559. (**F**) patient with ID 563. The red shade in the left panels denotes the hypoglycemia region (BG lower than 80 mg/dl), the green shade denotes the normoglycemia region (BG within 80 mg/dl and 180 mg/dl), and the yellow shade denotes the hyperglycemia region (BG higher than 180 mg/dl). Pink dashed lines denote the target glucose level (120 mg/dl) used in the reward calculations.

We systematically compare two offline RL algorithms, i.e., BCQ and TD3+BC, on patients in the OhioT1DM cohort 2018. The glucose trajectories in the original OhioT1DM dataset suggest that most patients experienced recurrent hyperglycemic events, while only one of the patients showed a short period of hypoglycemia (PID591). Informed by medical knowledge that higher insulin infusion can decrease the resulting glucose level, we broadened the permitted amount of the insulin infusion for RL agents by doubling the maximum action (insulin infusion rate) documented in the OhioT1DM dataset, without exceeding the clinically safe insulin dosage (Braithwaite et al., 2020). We trained the offline RL models for 10000 episodes and saved the best episode (defined as the episode when the highest reward was achieved for each algorithm and patient) for later evaluation (Fig. S7). Fig. 6 shows that both BCQ and TD3+BC RL agents provide much better insulin dosage plans for 5 patients, as indicated by the green (BCQ) and blue (TD3+BC) curves staying more frequently in the safe glucose region (BG level between 80 mg/dl and 180 mg/dl) denoted by the green shade, and leaning closer to the target glucose level (120 mg/dl) used to define the maximum reward of action. We also examined the time in range (Table S6), time above range (Table S7) and time below range (Table S8), corresponding to the frequency of glucose levels within 80 mg/dl and 180 mg/dl, below 80 mg/dl and above 180 mg/dl, respectively, for the glucose levels in these three methods. The results suggested that both BCQ and TD3+BC improved time in range in six patients, with TD3+BC showing slightly higher values. Consequently, the other two metrics, i.e., time above range and time below range, are lower in the offline models when compared to the data collected.

Interestingly, the statistical analysis of returns between the three methods on all six patients (Fig. S8) suggests that both offline RL agents are significantly better than the original offline data (p-value < 0.05), and there is no significant difference between BCQ agents and TD3+BC agents. In addition, we notice that the optimized insulin trajectories in BCQ seem noisier than those in TD3+BC because the VAE in BCQ reconstructs the variability of insulin infusion from the original offline dataset. While TD3+BC also imitates the distribution of action in the offline dataset with a behavior cloning term, TD3+BC supports tuning the strength of behavior cloning with a hyperparameter α, i.e., a higher α favors RL and a lower favors imitation, which was optimized during our training for patients. Although the original neural network architectures presented in TD3+BC and BCQ could potentially provide a better insulin plan compared to the offline dataset, we noted that increasing the depth of the actor network in TD3+BC and expanding the latent space dimension of VAE in BCQ resulted in a better glucose level in patients 563 and 575. This is probably because the non-linearity of glucose-insulin dynamics is more pronounced in these patients, given that the offline RL training does not require meal announcement or exercise announcement. We also found it beneficial to add a weighting factor $w_{KL}$ (Algorithm 1) to the Kullback–Leibler Divergence loss since training VAE in BCQ contributes to faster convergence in some patients. This may imply a variable balance between reconstruction loss and Kullback–Leibler Divergence loss when training with data from different patients.

**Fig. 7.**
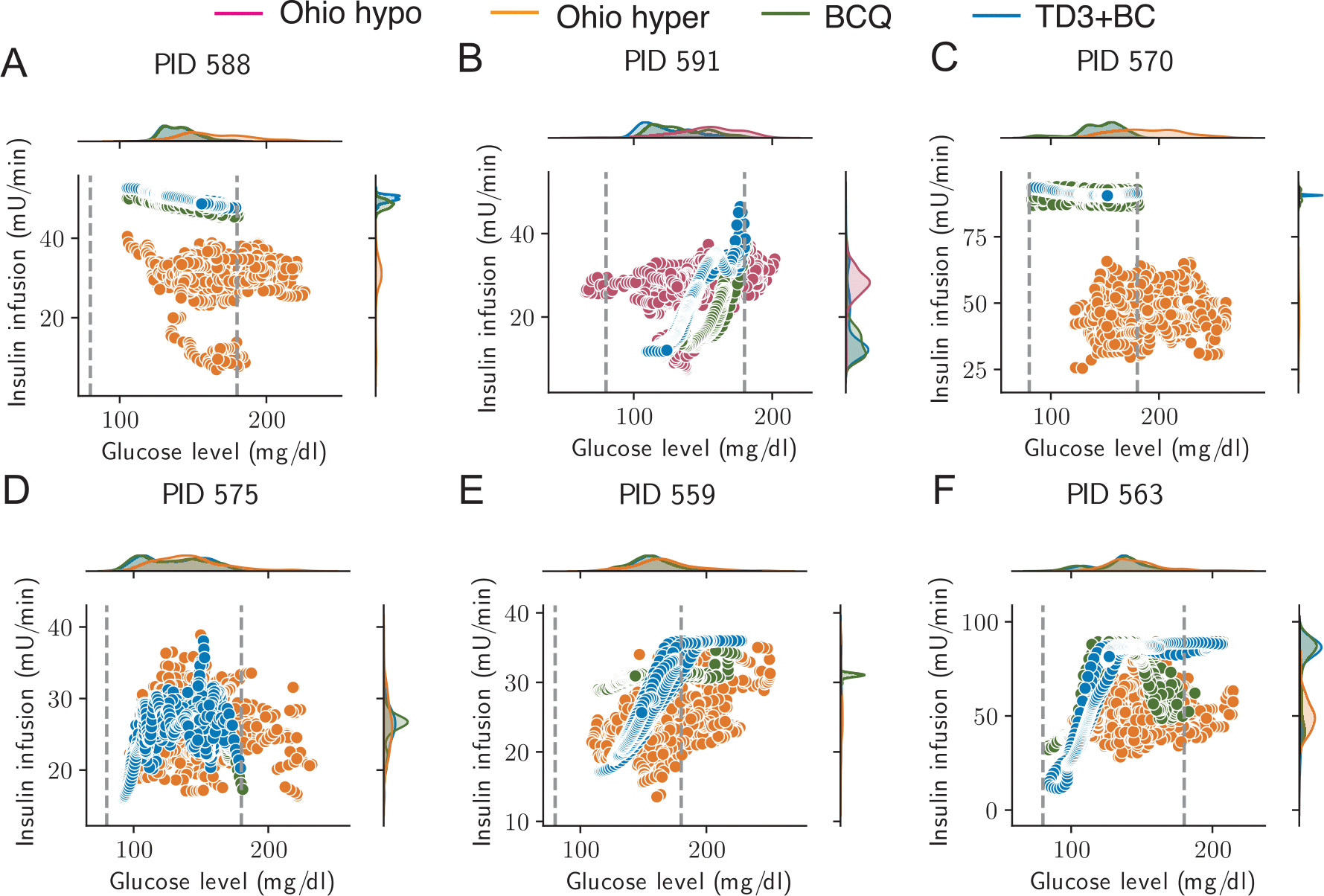
Deep offline reinforcement learning agents show a more coordinated insulin-glucose control pattern than the archived data in OhioT1DM. Scatter plots and probability density distributions (top for glucose level, right for insulin infusion) of insulin infusion-glucose level pairs in the OhioT1DM dataset (yellow) and the optimized offline RL (green and blue). (**A**) patient with ID 588. (**B**) patient with ID 591. (**C**) patient with ID 570. (**D**) patient with ID 575. (**E**) patient with ID 559. (**F**) patient with ID 563. Pink dots denote the patient experienced a period of hypoglycemia in the original OhioT1DM dataset. Yellow dots correspond to the patient who experienced frequent hyperglycemia in the original OhioT1DM dataset. Green dots denote the result of the BCQ agent. Blue dots denote the result of TD3+BC agent. Gray dashed lines represent the blood glucose cutoff of hypoglycemia (BG lower than 80 mg/dl) and hyperglycemia (BG lower than 180 mg/dl).

We also examined quantitative patterns of insulin infusion vs. glucose levels based on these three methods. The top panels in Fig. 7 suggest that the BCQ agents and TD3+BC agents successfully shift the glucose level distribution towards the defined safe range (BG level between 80 mg/dl and 180 mg/dl). Additionally, the right panels in Fig. 7 indicate that both the BCQ agents and TD3+BC agents learn to increase the overall insulin levels in patients with frequent hyperglycemia and lower insulin infusion in the patient with some hypoglycemic events, Fig. 7(B). It is noteworthy that offline RL agents exhibit greater synchronization between insulin administration and glucose levels, i.e., linear trends in the scatter plots of insulin infusion vs glucose levels in most patients, while the original offline dataset does not show such a trend. In addition, even though the offline RL agents have considerable freedom in choosing actions, the optimal agent consistently employs a strategy where the insulin dosage seldom reaches the maximum dose.

## 4. Discussion

Insulin is the mainstay of treatment for patients with type 1 diabetes mellitus and, oftentimes, long-standing type 2 diabetes mellitus to achieve good glycemic control (Shah et al., 2014). Overestimation of the necessary insulin dosage can be extremely dangerous and may lead to fatally low blood glucose levels below 70–80 mg/dl when measured by CGM, namely hypoglycemia, while an underestimated insulin dosage, leaving blood glucose above 180 mg/dl may result in hyperglycemia, which is believed to be responsible for micro- and macro-vascular diseases in the long run. In modern medicine, the use of insulin pumps along with continuous glucose monitors has made it easier, but requires significant resources and patient education. Fortunately, a closed-loop control system, also called an artificial pancreas (AP), which automates insulin infusion to maintain a consistently stable blood glucose level, undoubtedly relieves the burden of both patients and doctors and saves medical costs.

Specifically, we attempted to address a few challenges in designing next-generation APs with our framework, which effectively combines three key components to build a patient-specific artificial pancreas, simultaneously considering real-world data collected from wearable devices, i.e., meal intake, insulin infusion, and physical exercises. These important components are: (1) a real-world historical medical dataset, namely the OhioT1DM dataset, containing patient-specific glucose, insulin, meal intake, and exercise intensity; (2) a flexible ODE model defining glucose-insulin dynamics by systematically prioritizing two significant external factors affecting glucose levels, i.e., meal intake and physical exercise intensities; and (3) two offline RL algorithms without directly interacting with real patients’ metabolic environment. According to the Centers for Disease Control and Prevention, diabetic patients are advised to perform the evaluation of their treatment goals every 3 months or 6 months in clinical visits. To ensure broad applicability in the evaluation of diabetes management for patients, we designed this framework to operate on a monthly time scale without sacrificing its generalizability.

Despite the variations arising from distinct daily physical activity and insulin injection patterns among six different individuals, our model not only correctly predicts the hidden states that cannot be measured with current diabetes technology, but also accurately infers parameters governing the patient-specific Roy-Parker model. In addition, we also noted the consistent performance of the time-dependent SBINN across distinct runs. These findings collectively serve as robust evidence for the strong generalizability of our framework. More importantly, the uncertainty-qualified hidden parameters provide patient-specific clinical interpretations on how patients’ behavioral pattern shape their corresponding glucose-insulin dynamics. To design a safe artificial pancreas, we focused on developing reinforcement learning algorithms, which avoid dangerous data collection. Furthermore, we trained offline RL agents with two different offline RL algorithms, i.e., BCQ and TD3+BC, to automate insulin infusion and optimize the performance of the agent on the patient-specific ODE with the same glucose uptake and exercise intensity over time. Our results suggest that our offline RL agent has a better performance in terms of maintaining blood glucose levels within the safe range, compared to the self-operated insulin infusion by patients themselves. This implies that an agent trained by offline RL could learn a better insulin dosage depending solely on past glucose level sequences without meal or exercise announcements. This design could decouple the learning step of physiological parameters by SBINN from the training step of an optimal insulin agent by offline RL, hence leading to a cost-effective AP.

In spite of the improved glycemic control provided by our offline agent and minimal human intervention demanded by our framework, we can still identify possible improvements to the proposed framework, considering ODE model development, disease characterization, and data processing. As it is believed that there is both a time delay in the effect of insulin on glucose production and that on the glucose utilization (Sturis et al., 1991), it may be helpful to modify the ODE model to address the sluggish effects. Additionally, insulin can be categorized into fast-acting, intermediate-acting, and long-acting based on the timing of its action in the body. To account for this variability, we could extend the data preprocessing of external insulin and update the ODE model to allow for variations in insulin types (Evans et al., 2019; Zijlstra et al., 2018). To further enhance the closed-loop systems, it may be worthwhile to explore the incorporation of glucagon as a dimension of the action, a hormone that stimulates glucose production and can therefore increase plasma glucose levels (Peters & Haidar, 2018). By adding glucagon as an additional action, the RL agent will be able to explore the insulin action space with fewer constraints. In addition, glucose-insulin dynamics is a complex and multi-scale process being affected by other external factors, such as body weight variation, mental health (Anderson et al., 2001), drug-drug interaction (Triplitt, 2006). Due to missing body-weight information in the dataset, all patient-specific models assumed a nominal weight of 60 kg. Although this parameter can be absorbed into the glucose distribution volume, it remains a simplification that may underestimate inter-individual physiological variability. Future work will incorporate actual patient anthropometric data (e.g., body weight, BMI) to further enhance personalization and physiological fidelity. Fortunately, the proposed framework allows adaptation of the ODE form and the action space, hence enabling the incorporation of more external factors as forcing terms in ODE and extension of existing actions in offline RL agents. The accurate characterization of glucose afforded by this study at a monthly scale provides valuable insights for its potential adaptation to a smaller time scale, such as weekly, particularly for patients whose disease progresses at a faster rate than usual. We also note that systems biology-informed neural networks provide a promising opportunity to infer hidden dynamics in a biological system of interest, by leveraging the high expressivity of neural networks and primitive characteristics of the system in a low data availability scenario (Raissi et al., 2017). Whereas, in the context of healthcare, further multi-center clinical trials involving more participants will greatly improve the robustness of our framework. Another notable limitation of the current dataset is the imbalance between hyperglycemic and hypoglycemic events. Although one of the key motivations of this work is to improve the prevention of exercise-induced hypoglycemia, clinically significant hypoglycemia occurred predominantly in a single patient (PID 591), while the majority of participants exhibited mainly hyperglycemic excursions. As a result, the present study provides stronger validation for hyperglycemia management than for hypoglycemia prevention. Future studies incorporating datasets with a broader distribution of hypoglycemic episodes – particularly during and after physical activity – will be essential for fully evaluating and refining the framework’s ability to anticipate and prevent low-glucose events.

In summary, by seamlessly integrating real-world data from wearable sensors, time-dependent systems biology informed neural networks, and deep offline RL algorithms, we have developed a universal framework that could shed light on the patient-specific digital twin designs where adequate biological numerical models are established and enough medical data from sensors are available. In the context of type 1 diabetes, we found that SBINN could successfully infer the hidden physiological parameters governing glucose-insulin dynamics and accurately reconstruct the corresponding glucose trajectories at the patient’s level. In terms of deployability, both the SBINN and the offline RL controller operate in inference-only mode and therefore have low computational overhead. Inference can be performed in milliseconds on embedded processors used in current artificial pancreas devices, and future AP platforms are expected to include even more capable on-device AI hardware. Compression and optimization techniques (e.g., pruning, quantization) further support real-time implementation. Thus, the proposed framework is compatible with the computational requirements of modern and next-generation AP systems.Despite the limited number of patients, albeit with a complete dataset, we found that TD3+BC offline RL is not only more effective but also easier to tune than BCQ. Our framework shows translational potential in precision medicine by enriching digital models with extensive medical data collected from wearable devices (Raza et al., 2022; Venkatesh et al., 2022). For example, with a few slight modifications on the ODE model to address the insulin resistance of tissue, we could model and optimize insulin usage in patients with type 2 diabetes who develop insulin resistance (Cotero et al., 2022). Efforts in hardware designs of wearable sensors are also beneficial to our model development. For example, novel wearable devices are being designed to probe insulin and other metabolites that are impossible to quantify with existing techniques with a higher resolution (Poudineh et al., 2021; Wang et al., 2022). Furthermore, the rapid development in non-invasive wearable devices tremendously increases patients’ compliance in using wearable devices (Mitratza et al., 2022), which helps to reduce data sparsity with more frequent sensor readouts, and consequently improves the performance of our framework. These next-generation sensors will provide us with high-resolution and unprecedented data, which will significantly enhance the performance of our framework. Future artificial pancreas systems are expected to include more capable embedded processors and lightweight AI accelerators, which will support increasingly complex control algorithms. Because our framework operates entirely in inference mode and has a compact architecture, it is well suited for deployment on these next-generation devices.

## Supplementary Material

1

## Figures and Tables

**Table 1:** Parameter identifiability in the Roy-Parker model under four different scenarios.

Parameter	Identifibility			

	W and $I_{b}$ unknown	W known, $I_{b}$ unknown	W unknown, $I_{b}$ known	W and $I_{b}$ known

n	locally	locally	locally	locally
${\mathrm{Vol}}_{G}$	globally	globally	globally	globally
$p_{1}$	nonidentifiable	globally	globally	globally
$p_{2}$	locally	locally	locally	locally
$p_{3}$	nonidentifiable	nonidentifiable	locally	locally
$p_{4}$	nonidentifiable	nonidentifiable	locally	locally
$a_{1}$	nonidentifiable	locally	nonidentifiable	locally
$a_{2}$	locally	locally	locally	locally
$a_{3}$	nonidentifiable	locally	nonidentifiable	locally
$a_{4}$	locally	locally	locally	locally
$a_{5}$	nonidentifiable	nonidentifiable	locally	locally
$a_{6}$	globally	globally	globally	globally
$I_{b}$	nonidentifiable	nonidentifiable	–	–
W	nonidentifiable	–	nonidentifiable	–

W denotes the bodyweight of the patient, $I_{b}$ denotes the basal plasma insulin level. Details of other parameters can be found in Roy and Parker (2007).

## Data Availability

The OhioT1DM dataset used in the current study is publicly available on http://smarthealth.cs.ohio.edu/OhioT1DM-dataset.html.

## Code availability

The code and any intermediate files that are critical for the reproduction of this work will be released upon acceptance of publication and the approval of the data curator of OhioT1DM.

## Appendix A. Supplementary data

Supplementary material related to this article can be found online at https://doi.org/10.1016/j.smhl.2026.100633.
