# Supplementary material for: Patient-specific deep offline artificial pancreas for blood glucose regulation in type 1 diabetes
Source: Smart Health (Amst). Author manuscript; Available in PMC 2026 Mar 1. (PMC12945307; doi:10.1016/j.smhl.2026.100633)
Supplement: 1 [file NIHMS2141741-supplement-1.pdf]

7 Supplementary Material

Parameter	physiological meaning	reference value	time-dependent
n	rate of plasma insulin clearance	0.142 min^{-1}	Yes
Vol_G	glucose distribution space	117 dl	Yes
p_1	rate of insulin-independent glucose removal from the plasma space	0.035 min^{-1}	Yes
p_2	rate of insulin removal from the remote insulin compartment	0.05 min^{-1}	Yes
p_3	rate of insulin addition in the remote insulin compartment	2.8e-5 $\text{ml}/\mu\text{U} \cdot \text{min}^2$	Yes
p_4	*rate of insulin addition into the plasma from exogenous insulin	0.098 ml/min^2	Yes
a_1	*rate of exercise-induced hepatic glucose production	1.58e-3 $\text{mg/kg}\cdot\text{min}^2$	Yes
a_2	*rate of exercise-induced hepatic glucose consumption	0.056 min^{-1}	Yes
a_3	*rate of exercise-induced glucose uptake	1.95e-3 $\text{mg/kg}\cdot\text{min}^2$	Yes
a_4	*rate of exercise-induced glucose accumulation	0.0485 min^{-1}	Yes
a_5	rate of exercise-induced plasma insulin depletion during recovery period	1.25e-3 $\mu\text{U/ml} \cdot \text{min}$	Yes
a_6	rate of exercise-induced plasma insulin repletion during recovery period	0.0108 min^{-1}	Yes
u_{1b}	exogenous insulin infusion rate to maintain I_b	patient-specific	No
W	body weight	60 kg	No

Table S1: **Nomenclature and physiological meanings of patient-specific parameters in the Roy-Parker model**. Physiological meanings marked with * were not explained in the original publication, hence standing for the interpretation given the ODE form.

Patient record example

```
<patient id="563" insulin_type="Humalog 200">
  # CGM
  <glucose_level>
    <event ts="28-10-2021 00:01:00" value="231">
    ...
    <event ts="08-11-2021 08:14:00" value="145">
  </glucose_level>
  # basal insulin
  <basal>
    <event ts="28-10-2021 00:00:00" value="1.2"/>
    ...
    <event ts="01-11-2021 18:30:00" value="0.9"/>
  </basal>
  # temp basal insulin, not available for this patient
  <temp_basal>
  </temp_basal>
  # bolus insulin
  <bolus>
    <event ts_begin="29-10-2021 11:36:37" ts_end="29-10-2021 11:36:37" type="square dual" dose="3.6" bwz_carb_input="16"/>
    ...
    <event ts_begin="07-11-2021 19:43:37" ts_end="07-11-2021 11:36:37 type="normal" dose="3.2" bwz_carb_input="12"/>
  </bolus>
  # heart rate
  <basis_heart_rate>
    <event ts="28-10-2021 00:01:00" value="100"/>
    ...
    <event ts="08-11-2021 00:21:00" value="79"/>
  </basis_heart_rate>
  ...
  ...
  ...
</patient>
```

Figure S1: **A pseudo example of a patient's record in the OhioT1DM dataset showing the structure of the tabular data**.

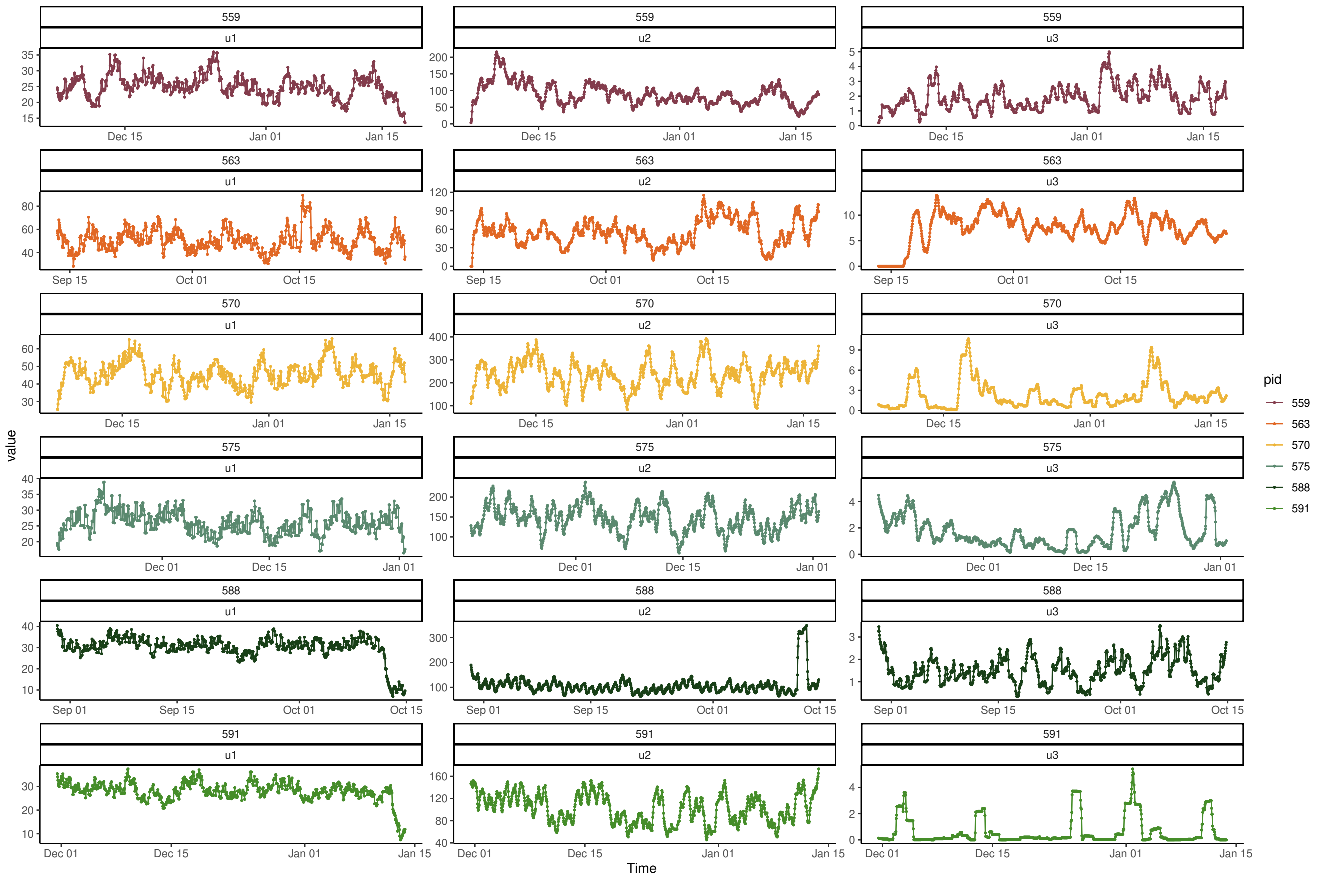

Figure S2: **External inputs extracted from patients' record in the OhioT1DM dataset**. u_1 denotes insulin infusion; u_2 denotes carbohydrate intakes; u_3 denotes exercise intensity.

Patient ID	Time series duration (hrs)
559	1007
563	1092
570	968
575	1092
588	1093
591	1063

Table S2: **Preprocessed OhioT1DM dataset for cohort 2018.** Note that the time series is trimmed such that the resulting time series starts and ends at the time stamps when the time series of different sources are available from wearable devices. Hence, all time series durations are slightly less than 1344 hours (hrs, equivalently 8 weeks).

Algorithm 1 Batch constrained Q-learning algorithm.

Require: Batch $\mathcal{B}$, horizon T, target network update rate τ, mini-batch size N, max perturbation Φ, weighting factor w_{KL}, number of sampled actions n, minimum weighting λ. Initialize Q-networks $Q_{\theta_1}, Q_{\theta_2}$, perturbation network ϵ_ϕ, and VAE $G_\omega = \{E_{\omega_1}, D_{\omega_2}\}$, with random parameters $\theta_1, \theta_2, \phi, \omega$, and target networks $Q_{\theta_1'}, Q_{\theta_2'}, \epsilon_{\phi'}$, with $\theta_1' \leftarrow \theta_1, \theta_2' \leftarrow \theta_2, \phi' \leftarrow \phi$. $t \leftarrow 0$.

while $t < T$ **do**

  Sample mini-batch of N transitions (s, a, r, s') from $\mathcal{B}$

  $\mu, \sigma = E_{\omega_1}(s, a), \tilde{a} = D_{\omega_2}(s, z), z \sim \mathcal{N}(\mu, \sigma)$

  $\omega \leftarrow \text{argmin}_\omega \sum (a - \tilde{a})^2 + w_{KL} D_{KL}(\mathcal{N}(\mu, \sigma) || \mathcal{N}(0, 1))$

  Sample n actions: $\{a_i \sim G_\omega(s')\}_{i=1}^n$

  Perturb each action: $\{a_i = a_i + \epsilon_\phi(s', a, \Phi)\}_{i=1}^n$

  Set value target y

  $\theta \leftarrow \text{argmin}_\theta \sum (y - Q_\theta(s, a))^2$

  $\phi \leftarrow \text{argmax}_\phi \sum Q_{\theta_1}(s, a + \epsilon_\phi(s, a, \Phi)), a \sim G_\omega(s)$

  Update target networks: $\theta_i' \leftarrow \tau\theta + (1 - \tau)\theta_i'$

  $\phi' \leftarrow \tau\phi + (1 - \tau)\phi'$

  $t \leftarrow t + 1$.

end while

Hyper-parameter	Value
Optimizer	Adam (77)
Learning Rate	10^{-3}
Batch Size	512
Normalized Observations	Yes
Gradient Clipping	No
Discount Factor	0.99
Target Update Rate (τ)	0.005
Exploration Policy	$\mathcal{N}(0, 0.1)$

Table S3: **Hyper-parameters used in BCQ.**

Algorithm 2 TD3+BC algorithm.

Initialize critic networks $Q_{\theta_1}, Q_{\theta_2}$ and actor network π_ϕ with weights ϕ
Initialize target networks $\theta'_1 \leftarrow \theta_1$, $\theta'_2 \leftarrow \theta_2$, $\phi' \leftarrow \phi$
Initialize replay buffer $\mathcal{B}$ and regularization constant λ
for t = 1 → T **do**
    Select action $a = \pi_\phi(s) + \epsilon$, where $\epsilon_t \sim \mathcal{N}(0, \sigma)$ and observe reward r and next state s'
    Store transition (s, a, r, s') in $\mathcal{B}$
    Sample minibatch of N transitions from $\mathcal{B}$
    $\tilde{a} \leftarrow \pi_{\phi'}(s') + \epsilon$, $\epsilon \sim \text{clip}(\mathcal{N}(0, \tilde{\sigma}), -c, c)$
    $y \leftarrow r + \gamma \min_{i=1,2} Q_{\theta'_i}(s', \tilde{a})$
    Update critics $\theta_i \leftarrow \text{argmin}_{\theta_i} N^{-1} \sum (y - Q_{\theta_i}(s, a))^2$
    if $t \mod d$ **then**
        Update ϕ by the deterministic policy gradient:
        $\nabla_\phi J(\phi) = \frac{\lambda}{N} \sum_{i=1}^{N} \nabla_a Q_{\theta_1}(s, a)|_{a=\pi_\phi(s)} \nabla_\phi \pi_\phi(s) + \nabla_\phi (\pi_\phi - a)^2$
        Update target networks:
        $\theta'_i \leftarrow \tau\theta_i + (1 - \tau)\theta'_i$
        $\phi'_i \leftarrow \tau\phi_i + (1 - \tau)\phi'_i$
    end if
end for

Hyper-parameter	Value
Optimizer	Adam (77)
Learning Rate	3e-4
Batch Size	512
Normalized Observations	Yes
Discount Factor	0.99
Target Update Rate (τ)	0.005
Policy noise	0.2 or 0.4
Policy noise clipping	1
Policy update frequency	2
Action space augmentation	2
Critic hidden dim	256
Critic hidden layers	2
Critic activation function	ReLU
Actor hidden dim	256
Actor hidden layers	2
Actor activation function	ReLU
TD3+BC specific regularization constant α	1 or 10

Table S4: **Hyper-parameters used in TD3+BC.**

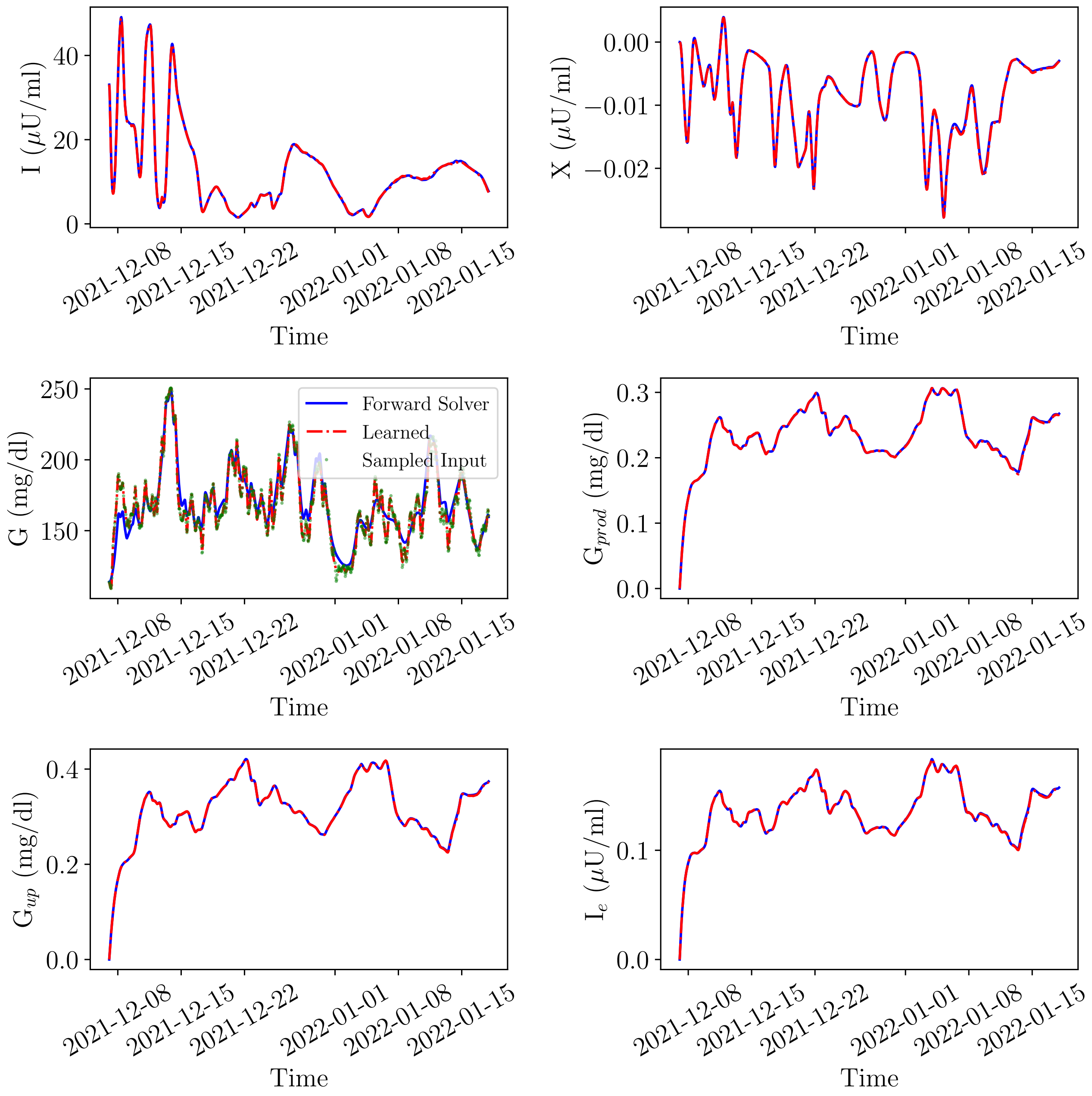

Figure S3: **Prediction of 6 state variables in the Roy-Parker model using SBINNs patient with ID 559**.

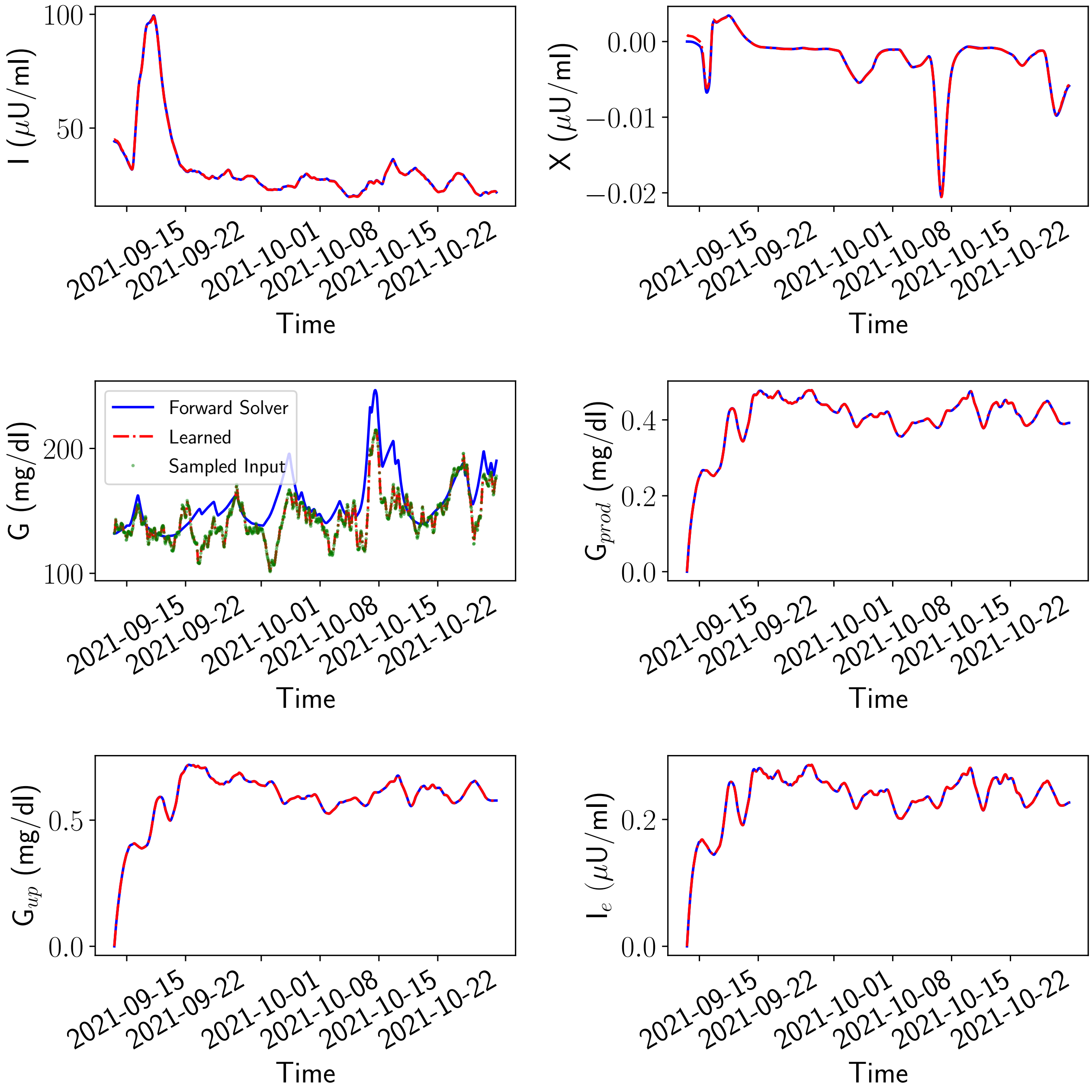

Figure S4: **Prediction of 6 state variables in the Roy-Parker model using SBINNs patient with ID 563**.

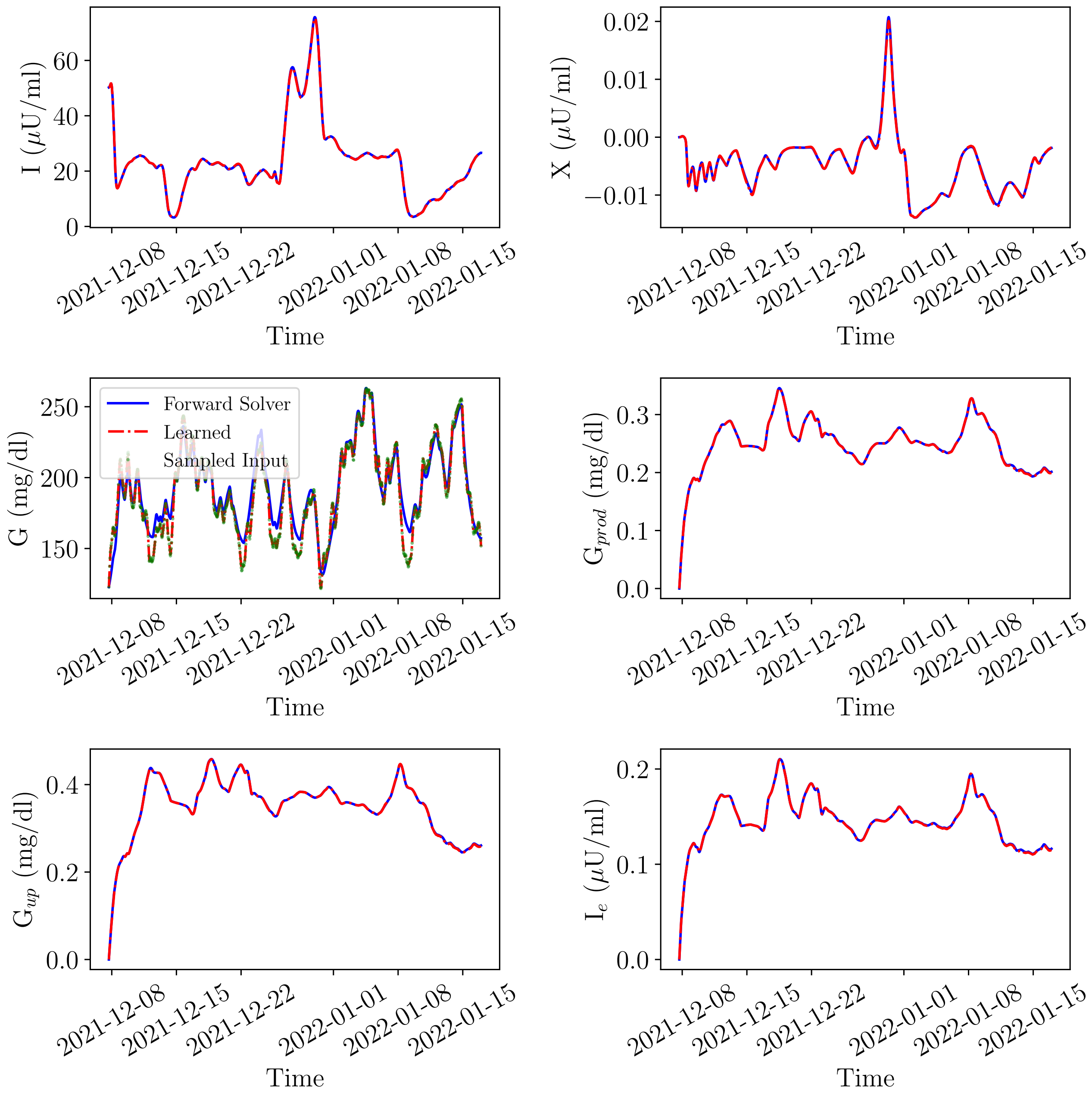

Figure S5: **Prediction of 6 state variables in the Roy-Parker model using SBINNs patient with ID 570**.

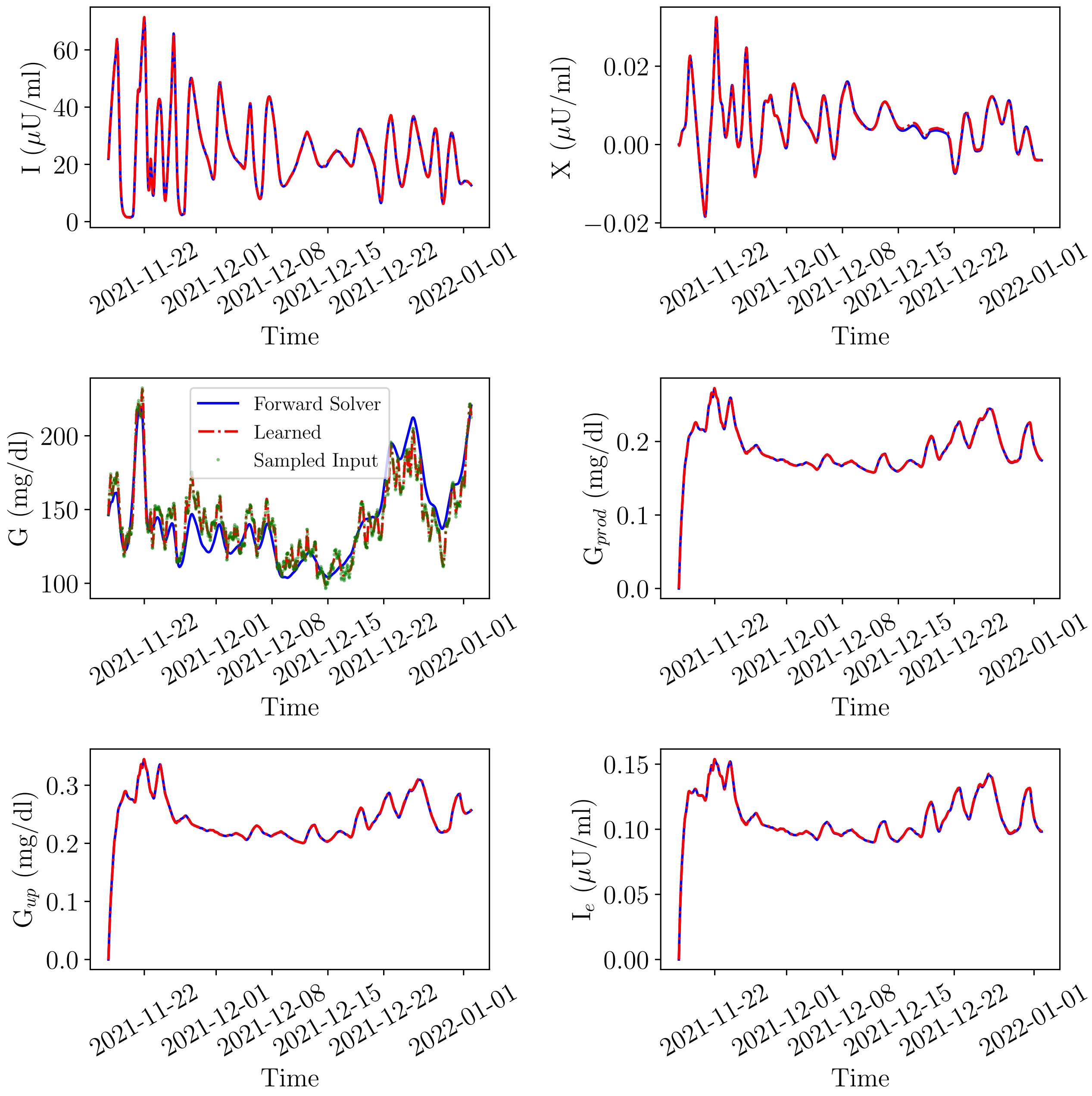

Figure S6: **Prediction of 6 state variables in the Roy-Parker model using SBINNs patient with ID 575**.

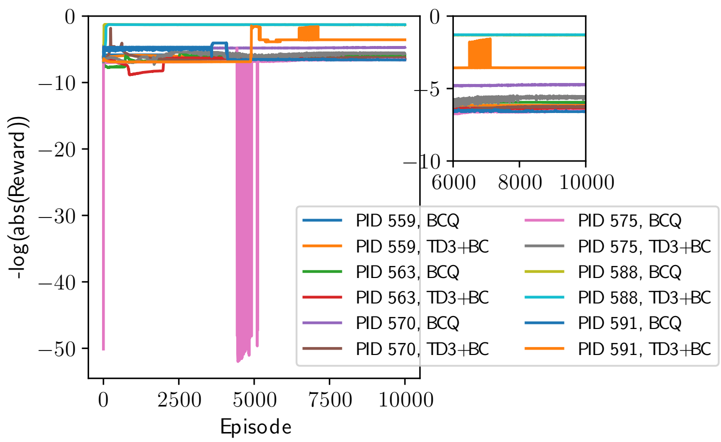

Figure S7: **RL training loss for 6 patients**. Inset shows the zoom-in figure of last 4000 episodes.

Patient ID	OhioT1DM	BCQ	TD3+BC
559	-1.760743e+06	-655134.632 **(+62%)**	-832684.556 **(+52%)**
563	-3.608720e+05	-202049.464 **(+44%)**	-201322.133 **(+44%)**
570	-4.928174e+06	-22237.888 **(+99%)**	-7309.135 **(+99%)**
575	-6.390143e+05	-1131.551 **(+99%)**	-3722.429 **(+99%)**
588	-1.628487e+06	-14719.126 **(+99%)**	-3654.431 **(+99%)**
591	-6.621690e+05	-11243.534 **(+98%)**	-3689.460 **(+99%)**

Table S5: **Reward comparison between RL based agents and original OhioT1DM dataset for 6 patients.** Values in parentheses denote percentage of reward increment in RL agents with respect to OhioT1DM.

Patient ID	OhioT1DM	BCQ	TD3+BC
559	0.7537	**0.8928**	0.8669
563	0.9396	**0.9698**	**0.9698**
570	0.4163	0.9938	**0.9979**
575	0.9121	**1.0000**	0.9991
588	0.7447	0.9963	**0.9991**
591	0.8542	0.9972	**0.9991**

Table S6: **Time in range comparison between RL based agents and original OhioT1DM dataset for 6 patients.** Higher means better performance. Bold denotes row-wise highest value.

Patient ID	OhioT1DM	BCQ	TD3+BC
559	0.2463	**0.1072**	0.1331
563	0.0604	**0.0302**	**0.0302**
570	0.5837	0.0062	**0.0021**
575	0.0879	**0.0000**	0.0009
588	0.2553	0.0037	**0.0009**
591	0.1336	0.0028	**0.0009**

Table S7: **Time above range comparison between RL based agents and original OhioT1DM dataset for 6 patients.** Lower means better performance. Bold denotes row-wise lowest value.

Patient ID	OhioT1DM	BCQ	TD3+BC
559	0.0000	0.0000	0.0000
563	0.0000	0.0000	0.0000
570	0.0000	0.0000	0.0000
575	0.0000	0.0000	0.0000
588	0.0000	0.0000	0.0000
591	0.0122	0.0000	0.0000

Table S8: **Time below range comparison between RL based agents and original OhioT1DM dataset for 6 patients.** Lower means better performance.

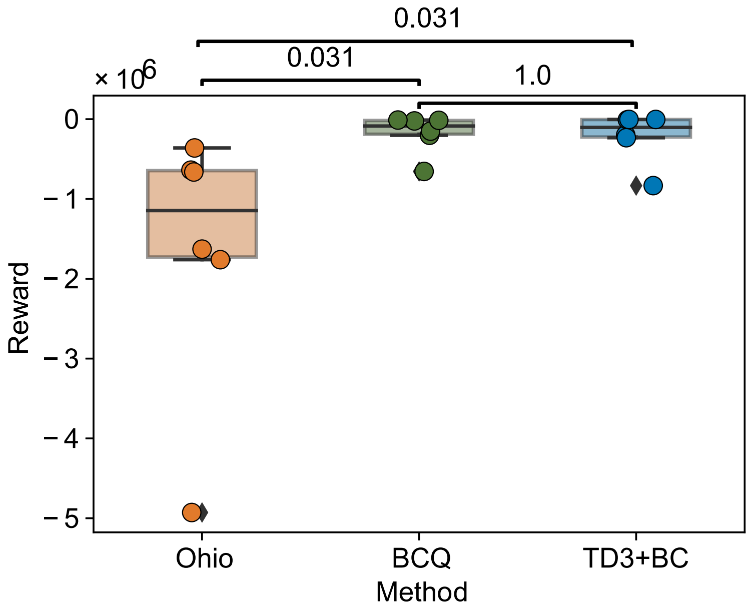

Figure S8: **Statistics of return comparison between RL based agents and original OhioT1DM dataset for 6 patients**. The paired Wilcoxon test was applied.
